# Euterpe music therapy methodology and procedure algorithms

**DOI:** 10.3389/fneur.2024.1443329

**Published:** 2024-10-28

**Authors:** Tommaso Liuzzi, Fiammetta D’Arienzo, Massimiliano Raponi, Paola De Bartolo, Miled Tarabay, Roberto Giuliani, Enrico Castelli

**Affiliations:** ^1^Unit of Neurorehabilitation, Bambino Gesù Children’s Hospital, IRCCS, Rome, Italy; ^2^Santa Cecilia Conservatory of Music, Rome, Italy; ^3^Euterpe APS Cultural Association, Rome, Italy; ^4^Health Directorate, Bambino Gesù Children’s Hospital, IRCCS, Rome, Italy; ^5^Department of Human Sciences, University Guglielmo Marconi of Rome, Rome, Italy; ^6^School of Music and Performing Arts, Holy Spirit University of Kaslik, Jounieh, Lebanon

**Keywords:** music therapy, Euterpe method, rehabilitation, children, sleep quality, parental distress, quality of life, cerebral palsy

## Abstract

**Introduction:**

As highlighted by the scientific literature, music therapy (MT) represents a significant non-pharmacological intervention within neurorehabilitation programs. MT offers benefits in the recovery process and enhances the quality of life for patients with neurodevelopmental disorders. A review of the literature reveals a lack of MT models focusing on real-time personalized composition using electronic music techniques. Furthermore, studies on MT conducted within a multisensory therapeutic context are limited. Recent literature reviews on MT in telerehabilitation have highlighted that the application of the Euterpe Method (EM) is complex due to limited technical information available and the combined background required for music therapists to replicate the EM protocol.

**Methods:**

This paper presents a manual which specifies the procedures and algorithms of the EM, developed during a research program conducted in a pediatric hospital in Italy. The prerogative of the EM is the use of procedures aimed at creating personalized therapeutic compositions within a multisensory environment.

**Discussion:**

The efficacy and resilience of the EM have been demonstrated in two experimental studies. The first focused on the use of telerehabilitation in children with developmental disorders, while the second involved hospitalized children with cerebral palsy.

**Conclusion:**

This study integrates medicine, neuroscience, and MT to develop personalized interventions in pediatrics, fostering collaboration among specialists and families, enhancing patient well-being, and opening new therapeutic perspectives, while ensuring the replicability of the EM approach.

## 1 Introduction

The use of music as therapy has ancient origins, initially intertwined with myths, cosmologies, and theories of the ancient world. In many myths and popular narratives, sound is often conceived as the primordial substance from which the universe originates. Early historical references include Egyptian medical papyri dating back to before 1,500 BCE, Book III of Plato’s Republic, and the Arab-Jewish medical tradition (1–8). Over time, musical therapies evolved from shamanic practices to tribal medicine and tarantism (9).

The development and formalization of music therapy (MT) began with the establishment of the first academic program at Michigan State University in 1944, followed by the creation of the National Association for Music Therapy in 1950 (10). This professionalization continued with the founding of the British Society for Music Therapy by Juliette Alvin in 1958, expanding the reach of MT in Europe. In 1985, in response to the increase in experiences and the growing proliferation of MT associations, the World Federation of Music Therapy (WFMT) was established. This organization has played a pivotal role in formalizing the discipline by defining ethical, deontological, and professional standards, as well as promoting a shared definition of MT at the international level (11). To support this process of disciplinary structuring, during the 9th World Congress of Music Therapy in 1999, five main models of MT practice were formally recognized, developed by authoritative figures in the scientific community such as Bonny (12), Priestley (13), Nordoff and Robbins (14), Benenzon (15), and Madsen (16).

With the evolution of MT methodologies, techniques have been further refined through the integration of neuroscientific knowledge and the use of new musical technological instruments. In this context, scientific reviews have highlighted that musical improvisation is a prevalent strategy in numerous MT methodologies, as it facilitates emotional expression and increases therapeutic effectiveness (17–19).

These characteristics are identifiable in the approaches of Priestley and Nordoff-Robbins, whose therapeutic models closely align with our clinical practice, being based on musical improvisation and the creation of compositions specifically tailored to the patient’s needs (20). In parallel, the work of Anne Riordan and Kenneth E. Bruscia has introduced dance as a complementary therapeutic element in musical improvisation, demonstrating how the integration of this dimension promotes improvements not only in emotional and social skills but also in the patient’s cognitive and motor abilities (21).

Although the use of musical improvisation promotes creative expression, it introduces a degree of unpredictability that may not be suitable for all patients. Indeed, the absence of a predictable structure can make it challenging to maintain a clear therapeutic focus, potentially compromising the effectiveness of the intervention.

Bruscia has also highlighted, from an applied perspective, a range of risks and limitations associated with the patient. These include the need for adequate cognitive and sensorimotor skills to engage in musical improvisation. There is also the risk of adverse psychological reactions to music, such as hallucinations, difficulties in auditory processing, and undesirable physical responses. Other side effects include withdrawal, obsessions, compulsions, and sensory overload. Lastly, the author advises paying attention to interpersonal dynamics that may be threatening to the patient (22).

To prevent the risks associated with improvisation in MT, it is essential to tailor sessions according to the patient’s psychophysical condition, ensuring safe participation and preventing frustration. It is equally important to carefully monitor adverse psychological reactions and side effects, selecting musical materials with precision, and adjusting the intensity of the treatment. The therapist must meticulously consider the patient’s sociocultural context, respecting musical traditions to prevent cultural dissonance that could undermine the effectiveness of the therapeutic intervention. Contextually, the integration of innovative musical technologies can significantly enhance the patient’s auditory processing and mitigate the impact of potentially disruptive sound stimuli.

Scientific reviews highlight how such technologies can significantly enhance the therapeutic potential of MT. Consequently, these technologies applied to electronic composition can be tailored to meet the specific needs of patients (23–27). Supporting this, Farnan defines composition as “custom designed” music (25). Jones supports the value of composition, emphasizing the need for further research on best-practice models for clinical objectives (26). Barta et al. demonstrate the value of real-time music composition in the rehabilitation of patients with Parkinson’s disease (27). These findings are supported by the review conducted by Vinciguerra et al., which demonstrates that MT improves motor function and cognitive abilities in patients diagnosed with Parkinson’s and Alzheimer’s diseases, as well as stroke. This result emphasizes the significance of musical temporal structures in enhancing motor coordination and memory. In addition to its applications in neurodevelopmental disorders, MT has also demonstrated significant non-pharmacological benefits in managing chronic neurological diseases such as multiple sclerosis. The authors highlighted the role of MT in enhancing emotional well-being and cognitive functions in multiple sclerosis patients, demonstrating its broad therapeutic potential in neurorehabilitation (28).

Another aspect considered in our study is the limited number of research studies on MT in a multisensory context. Although some reviewed studies report sound or compositional interactions in relation to the environment, there are no specific references concerning the interactions between the environment, musical activities, and intervention methodologies (29–34). Marti et al. evaluated the effectiveness of MT therapeutic protocols in a multisensory environment for the treatment of dementia (29). Maseda et al. and Latham et al. further explored the effectiveness of non-pharmacological interventions in multisensory environments on patients with dementia, using different therapeutic approaches. Maseda et al. explored the use of unstructured stimuli autonomously chosen by patients, while Latham et al. evaluated Simard’s Namaste Care program, which combines scents, lighting, sensory objects, and music to create a relaxing atmosphere, employing personalized playlists only when necessary (30, 31). However, the studies present methodological limitations, particularly regarding the interaction between multisensory stimulation and the musical component. Lee and Li reported a school study focused on the impact of therapeutic musical activities in a multisensory environment for children with multiple disabilities, aimed at promoting positive emotional development (32). Cappelen and Andersson introduced an interactive multisensory environment that integrates music composition, design with the therapeutic principles of music, MT, and sensory integration to promote health through technology (33). Challis et al. presented two case studies that question current trends in the design and allocation of resources for multisensory environments, emphasizing the creation of functional spaces rather than simple technology aggregates (34).

The literature review has identified significant biases in the field of MT, including the limited application of real-time personalized electronic composition techniques.

Moreover, most existing approaches focus on environmental setup and the use of musical instruments, without fully considering the role of the environment as a sensory amplifier (2, 12, 15, 22). When used correctly, the environment could function as a sensory “mixer,” modulating and enhancing the effectiveness of the intervention.

Finally, although the Euterpe Method (EM) has demonstrated its efficacy in the context of both neurorehabilitation and telerehabilitation (35, 36), subsequent reviews have raised both growing interest and significant criticisms (37–48). Specifically, Paterson et al. have emphasized the importance of implementing telerehabilitation interventions based on MT to address new challenges in modern medicine (39). Concurrently, Le Vu et al. have highlighted the complexity of implementing EM, as it requires interdisciplinary training for music therapists, an aspect that currently limits the method’s replicability and widespread adoption (41).

To address the identified biases, this article proposes targeted interventions aimed at enhancing replicability and interdisciplinary training for music therapists through the use of original EM algorithms and procedures. These algorithms include EM Hospital-based (providing specific clinical goals for each patient), EM Active (live music, real-time compositions, improvisation, interactive workshops), EM Receptive (listening to personalized compositions and electronic music), and EM Telerehabilitation (remote delivery of MT). Furthermore, integrating a multisensory space with compositional activities represents an innovative strategy to amplify MT’s effectiveness, improving the interaction between the environment and therapeutic intervention.

The integration of neuroscience, music, and technology not only opens new perspectives for personalized and clinically optimized therapeutic interventions, but also lays the groundwork for structured innovation in pediatric rehabilitation.

### 1.1 The Euterpe method

The methodology referred to as Euterpe has a dual foundation. Culturally, Euterpe is one of the nine Muses of Greek mythology, the daughter of Zeus and Mnemosyne, and the protector of music. The name, derived from the Greek words ἐυ (good) and τέρπεω (pleasure), means “she who gives joy.” Pragmatically, it reflects the intent to develop a methodological approach tailored to the individual needs of patients and therapeutic goals, in close collaboration with the music therapist and the medical team.

EM is designed for children with neurodevelopmental disorders, actively involving parents, typically the mother, during therapy sessions.

EM uses original, customized *Compositional Sound Interventions* (CSI) and structured therapeutic activities based on specific algorithms. Music and CSI form the foundation for creating *Personalized Therapeutic Compositions* (PTCs), which constitute the core of EM therapy.

Therefore, PTCs can be defined as a series of articulated processes of specific sound stimuli, technically processed and adapted to the patient’s individual needs during therapy. These sound processes are simultaneously associated with multisensory stimulation within an enriched environment.

In order to delve deeper into the process of PTC realization and implementation, we feel it is essential to present a brief excursus.

As widely acknowledged, music has a significant impact on mood and emotions. This effect occurs because sound stimuli exert a sensory and psychological response in each individual, inducing a corresponding emotional reaction (49–51).

In support of the above, anthropologist Alan Parkhurst Merriam identifies 10 functions for music, representing opportunities to express emotions, thoughts and ideas (5).

Numerous musicological studies affirm that a musical work is structured as a series of more or less lengthy musical segments, arranged in a functional “hierarchy” that determines their role and importance (52, 53).

These studies suggest an affinity between personalized musical composition and the creation of a therapeutic context that resonates with the patient’s experiences, needs, and aspirations.

In the creation of PTCs, the compositional sound material derived from the patient’s “positive” reactions assumes a “*hierarchical perceptual function*.” This function reflects the patient’s ability to interpret and respond to musical stimuli according to their subjective perceptions, which are intimately tied to their somatosensory condition and psychophysiological state.

The implementation of the PTC develops through an articulated temporal flow, composed of “sound cells,” compositional structures, and specific timbres. These components impart a dialogic quality to the PTC, in which sound sequences alternate in a rhythm of “coming and going,” presenting the composition as a sound dialog of questions and answers, clearly distinguishing a “before” and “after.” This compositional development does not conform to predetermined musical forms, as it is solely guided by the therapeutic question: *“Does this intervention improve the patient’s quality of life?”* However, it is noteworthy, upon subsequent musicological analysis, that PTCs reveal similarities with pre-existing musical forms and musicological philosophies. This highlights how musical perception is an acquired anthropological construct, reflected in specific compositional processes. Below are examples that overlap with the compositional techniques of PTCs.

During EM therapy, a relevant example is guiding the patient to listen to silence and the sounds produced by the environment. This approach aligns with John Cage’s philosophical idea of 4′33″, a composition consisting entirely of silence, where the listener is confronted only with environmental sounds, the only tangible source of “music.” The absence of sound in favor of casual and incidental sounds (such as the audience’s breathing or noises inside and outside the auditorium) becomes the new sound material (54). Even more significant is Cage’s composition Imaginary Landscape No. 4, performed with 12 radios and 24 performers, where the listening result is always unique and unrepeatable due to the simultaneous overlap of sounds, words, and random noises from radio stations. This concept is similar to that of PTCs, with the distinction that every sound variation produced by the patient in the multisensory environment in which therapy takes place is recorded and played back as a new compositional incipit.

A similarity between the compositional structure of PTCs and Lerdahl and Jackendoff’s Generative Theory of Tonal Music lies in the use of generative principles to organize and interpret music. This theory, based on the hierarchy of tensions and relaxations, as well as processes of continuity and discontinuity, finds a parallel in PTCs, where sound events like the mother’s prosodic voice and the patient’s reactions alternate in a rhythmic loop, creating dynamic contrasts between tension and relaxation (55–57).

Additionally, minimalist styles from composers such as Terry Riley, Steve Reich, Philip Glass, and John Adams can be identified, as they conceptualize a “gradual musical process” based on repetition, variation, and structural prolongation of rhythmic and melodic segments (58).

Similarly, the cluster technique employed by György Ligeti (59) can be recognized. This approach consists of modifying through “addition” or “subtraction” complexes of chromatic and diatonic sounds, as nuances of a sound spectrum, creating micro-polyphonies that alternate in a compositional continuum.

Finally, the choice and use of electronic music for PTCs in EM therapy. It is important to note that electronic music originates from recording, listening, and developing electroacoustic instruments.

In fact, electronic composition is directly related to the sound material, unlike traditional composition, which uses symbolic writing (notation) to represent the gestures to be performed on a musical instrument to play a song.

The evolution of electronic composition has traversed research sector, progressing through stages such as phonography (a means of capturing and reproducing sounds while keeping them identical), radiophony (enabling real-time live transmissions over long distances), and electric and electronic lutherie (offering innovative musical instruments). The evolution of computers has made it possible to combine the characteristics of phonography, radiophony, and electronic lutherie into electronic composition. Finally, ongoing developments in computer technology, research, and new tools have consistently advanced the sector of electronic music, drawing attention and encouraging scientific studies across a wide range of disciplines, including MT.

Electronic music was born in Cologne in 1950 by Herbert Eimert. The growing interest in this domain among scholars led to the establishment of significant research centers, notably: the Studio di Fonologia Musicale, founded by Bruno Maderna and Luciano Berio in Milan in 1955 (60); the Équipe de mathématique et d’automatique musicales (EMAMu), founded by Xenakis in 1966 (61); and the Institut de coordination acoustique/musique (IRCAM), established by Boulez in Paris in 1975 (62).

Key advantages of using electronic music in MT include the creation of therapeutic orchestral scores with virtual instruments and real-time interaction with the patient. Additionally, electronic music enables customization of sounds and synthesis effects tailored to the patient’s psychophysiological conditions and clinical objectives.

Examining the context of the previous discussion regarding the scientific-musical excursus that encompasses the emerging forms derived from PTC and the importance of electronic composition, a reflection arises on the context in which MT interventions take place.

The spaces dedicated to musical listening in various contexts are continuously evolving, influenced by research on acoustic techniques. It is essential to deepen the understanding of the environments in which MT activities occur, as these contexts can significantly impact the effectiveness of the MT sessions.

According to Altenmüller and Schlaug, music is not merely an auditory input but represents one of the most enriched experiences in terms of emotional, sensory, and cognitive dimensions, influencing and modulating the activity of brain regions involved in multisensory and motor integration, including frontal, parietal, and temporo-occipital regions (63). This multisensory brain activation is closely associated with the phenomenon of neuroplasticity. Neuroplasticity, or cortical remapping, involves reconnecting previously disconnected brain areas through new neural connections or strengthening existing ones. This process occurs in response to learning new skills, memory formation, recovery from injuries, and experiences of sensory deprivation or environmental enrichment (64).

Based on these studies (63, 64), the environment is configured as a determining factor that influences and contributes osmotic ally to the effectiveness of therapeutic sessions, facilitating the interaction between external stimuli and the patient’s psychophysiological processes.

There is limited research investigating the correlation between sound and environment in MT practice. However, examined studies have shown that the acoustic environment can modulate individuals’ emotional and behavioral responses, thus impacting MT effectiveness (65, 66).

Our research aims to implement sound activities into MT, recognizing that these activities extend beyond mere sound production, engaging all the senses. According to a study by Lee and Li (32), multisensory musical experiences can promote greater participation and emotional engagement, thereby enhancing the therapeutic effects of music.

Furthermore, it is important to consider the role of physical and social environments in facilitating or hindering the therapeutic process. Research by Wigram, Pedersen, and Bonde has demonstrated that the design of spaces dedicated to MT can affect group dynamics and perceptions of social support, thereby improving the overall efficacy of the sessions (67).

Based on the above considerations, EM sessions are conducted in a specially designed environment known as the “Synesthesia Room” (SR), equipped with specific materials and instruments aimed at providing complex and appropriate sensory stimulation. The therapeutic activity, referred to as “Sound in Multisensory Stimulation,” integrates the environment through the control and customization of sound stimuli, facilitating the simultaneous overlay of multiple sensory stimuli. The goals of the therapy are to improve, motivate, create, implement, and learn behaviors in the socio-communicative, affective-relational, psychomotor, cognitive, and neuropsychological domains (68).

Currently, the EM is employed in various clinical settings, including neurorehabilitation units, oncology departments in the Middle East and Europe, and MT associations.

### 1.2 The Euterpe method and the environment

The SR is an environment specifically designed and structured for EM therapy, which employs multisensory integration simultaneously with MT activities, considering it an integral part of the therapy.

The environment and sensory have been the subject of study and research since the 19th century; however, it was in the 1970s that the first rooms equipped for sensory stimulation through visual, auditory, tactile, and olfactory modalities (e.g., the Snoezelen room) were designed and tested (69).

Recent studies have shown that sensory integration engages multiple brain regions, such as the neocortex, basal ganglia, thalamus, and cerebellum. Within this framework, the cerebellum plays a central role in sensory processing, while the anterior motor cortex orchestrates motor programming. Disruptions—whether structural, metabolic, or functional—within these regions, or along their neural pathways, can profoundly compromise sensory processing, contributing to a spectrum of neurosensory dysfunctions (70, 71).

To date, clinical trials have focused on patients with intellectual and developmental disabilities or cognitive disorders such as dementia (70–74). As highlighted by Breslin et al., the therapeutic use of multisensory environments also aims at relaxation, pain management, improved attention, and the reduction of maladaptive behaviors (73).

Furthermore, from a musical perspective, the interaction between sound, environment, and tools has been a subject of scientific interest. Two prominent figures in this field are R. Murray Schafer and Aleksandr Skrjabin. Schafer is known for his studies on the “soundscape,” focusing on the relationship between sound and the natural and urban environment (75). Skrjabin, on the other hand, developed synesthetic theories linking music to colors and tactile sensations, opening new perspectives on the multisensory interaction of music (76). Both authors provide strong theoretical foundations for understanding how sound can be meaningfully integrated into the therapeutic environment and the patient’s experience.

Based on this research, EM therapy has developed therapeutic sound and compositional activities aimed at interacting synergistically with the SR environment. The environment itself is characterized by structural and sensory flexibility, allowing rapid adaptability to the specific needs of the patient.

A distinctive feature of the EM space is its adaptability and ease of configuration, making it suitable for various environments, including the patients’ home settings. The concept of “Mobile Architecture,” applied to the method, allows for the dynamic customization of the therapeutic environment. This flexibility not only optimizes the use of available space but also ensures that EM sessions can be seamlessly integrated into different therapeutic contexts, enhancing both the efficiency and quality of therapy.

This concept of “environmental resilience” has been discussed by Kaplan and Kaplan, who emphasized the importance of a therapeutic setting that can dynamically adapt to the individual needs and preferences of the patient (77).

Therapeutic interventions are designed in unimodal, multisensory, and cross-modal manners to maximize therapeutic efficacy. A unimodal approach may involve the use of a single sensory modality, such as vision or hearing, while a multisensory approach involves the simultaneous activation of multiple senses. Cross-modal stimulation, which engages the interaction between multiple senses, has been associated with enhanced brain plasticity and learning (78).

The therapeutic space of the SR is configured and personalized with specific materials and software to meet the individual needs of each patient, considering factors such as age, pathology, clinical condition, and specific therapeutic requirements (Supplementary 1). This personalization allows the patient to decontextualize the hospital environment, enabling the child to feel like the protagonist within the therapeutic setting. The design of the SR, grounded in robust scientific research, demonstrates that MT interventions in multisensory environments enhance the cognitive, emotional, social, and affective well-being of patients.

## 2 Materials and equipment

To ensure the proper conduction of EM MT sessions, the music therapist manages a range of devices and technological tools aimed at regulating sensory stimuli. It is important to note that the software specified is provided for informational purposes, and therefore, other programs may be used depending on expertise and specific usage needs. These tools include:

- Audio Management: A computer, using a Digital Audio Workstation, handles audio editing software such as Adobe Audition, Pro Tools, FMOD Studio, MPC Beats, Ableton Live, or similar programs. Audio playback is achieved through two active 2.1 systems, each composed of two tops with four speakers in a vertical array configuration and a subwoofer, totaling 1,200 W of maximum power. The setup is complemented by a 12-channel mixer, a 4 In/4 Out audio interface, dynamic and condenser microphones, as well as a sound pressure level meter (*cf.* Supplementary 2.1, 2.2).
- Video Management and Projection: Session recording and study are conducted using fixed and mobile cameras. Fixed cameras record in 4 K with HDR technology and a fixed 120° XY stereo microphone, while mobile cameras also allow 4 K recordings and are equipped with a mid-side stereo microphone. Projection equipment includes Full HD LED projectors and WVGA LED mini-projectors (*cf.* Supplementary 2.3).
- Lighting Management: Equipment comprises a lighting management station with DMX interface and RGBWA LED moving heads. Light management is carried out through a computer with ADJ MyDMX3 software to control color tones and light intensity (*cf.* Supplementary 2.4).
- Musical Instruments and Equipment: Various types of musical instruments are available for therapists and patients (*cf.* Supplementary 2.5), while olfactory stimulation is managed through essential oils and essence vaporizers. Additionally, a hammock is permanently installed to promote muscle relaxation and strengthen the patient-parent bond.

## 3 Methods

This study adopts a hermeneutic approach to deeply analyze the methodologies and procedural algorithms of the EM, integrating original research with a well-established scientific and historical context (79, 80). A rigorous qualitative review of the existing literature on MT practices was conducted, selecting sources of proven relevance and methodological robustness (81). The analysis incorporated data extracted from our previous studies and research as well as clinical reports (35, 36).

To contextualize the results, the historical development of MT was traced, positioning the EM within this broader framework. Key historical references have provided a solid theoretical basis for understanding the evolution and adaptation of therapeutic practices over time (82, 83). The investigation revealed that the EM fits into a historical tradition of therapeutic music use, with ancient roots spread across various cultures (84, 85).

The hermeneutic approach enabled a deep understanding of the experiences of patients and therapists with the EM through the interpretation of qualitative data (80). To ensure an inclusive and participatory method, the principles of Mertens’ transformative approach were integrated (86), actively involving patients, parents, and therapists in the research process. This approach fostered the empowerment of participants and promoted continuous dialog between researchers and the involved subjects, ensuring that their experiences and perspectives were fully integrated into the analysis.

The integration of historical elements and the triangulation of data sources ensured methodological rigor and high validity (87, 88). This combined approach not only facilitated a deeper understanding of the role of the EM in contemporary MT but also highlighted its potential to significantly improve patient well-being and promote the future development of the discipline.

### 3.1 Study design

This section analyzes the data from two previously published studies, aiming to provide a more detailed and comparative view of the effectiveness of EM in the context of intensive pediatric rehabilitation.

The first study, conducted by Bompard et al., was a prospective single-center study designed to evaluate the feasibility of EM-based telerehabilitation during the SARS-CoV-2 pandemic.

The inclusion criteria consisted of children with developmental delays under the age of 12 who had previously undergone personalized MT sessions prior to the pandemic. Exclusion criteria included the absence of participation in the EM MT pathway during the previous hospitalization.

Participants, regularly enrolled in intensive rehabilitation programs, were assessed using the Gross Motor Function Classification System (GMFCS) to determine their functional status (89). Evaluations were conducted remotely by the pediatric neurologist in collaboration with the parents, both before the start of the program and upon its conclusion. Informed consent was obtained from the parents for data collection and analysis (35). The second study, conducted by Liuzzi et al., was a prospective single-center experimental investigation aimed at evaluating the effects of the intervention on key aspects of the children’s and their parents’ lives.

Inclusion criteria comprised children aged 0 to 10 years with cerebral palsy (90), hospitalized for 3 to 4 weeks for intensive rehabilitation. Children with severe hearing impairments or deafness were excluded. Participants were randomly assigned to an experimental group, which received MT based on the EM, and a control group, which followed the rehabilitation program without MT. Motor functionality was assessed using the GMFCS (89), and outcomes were measured through the administration of parent-report questionnaires at T0 (study initiation) and T1 (after 3 weeks). Informed consent was obtained from the parents, and an independent evaluator, blinded to the randomization, examined the results (36).

### 3.2 Euterpe method procedures

EM’s distinctive prerogative lies in the use of four original algorithms, each designed to address specific therapeutic aspects with scientific precision.

**Figure 1:**
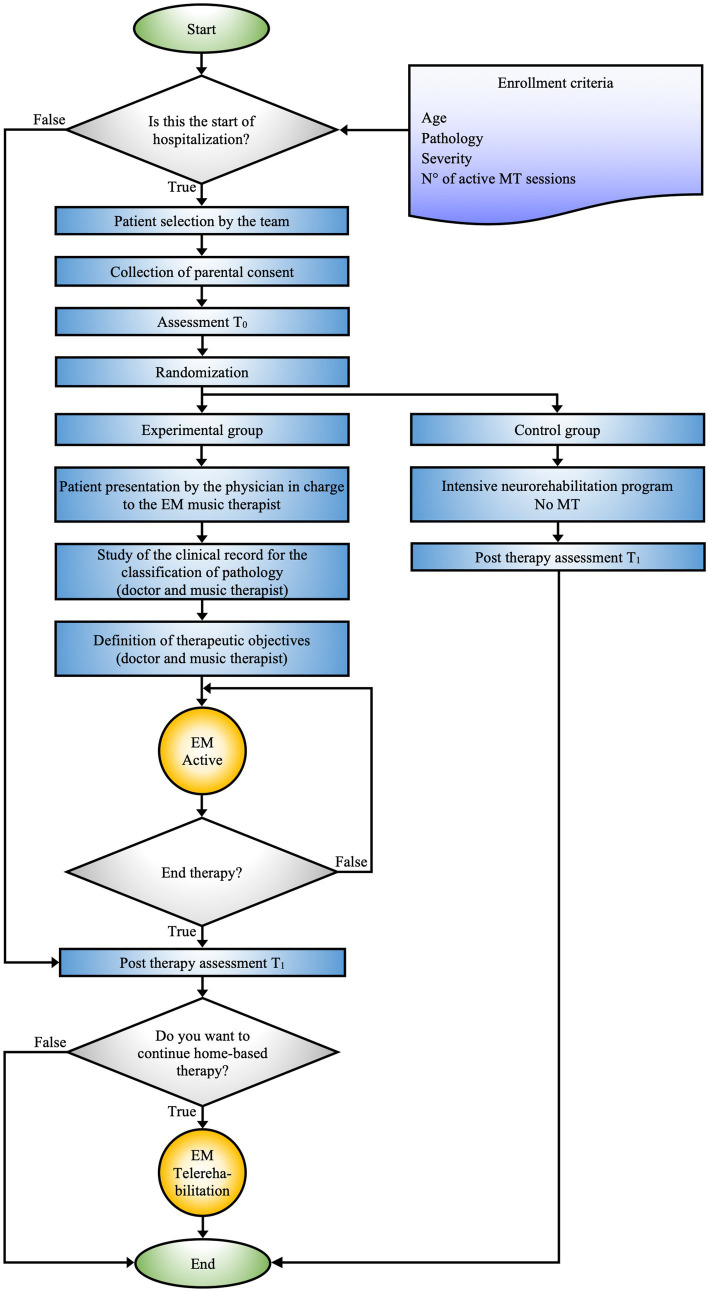
EM hospital-based schematic representation of the decision-making and therapeutic process adopted for patient management during hospitalization. The figure shows the integration between personalized MT and intensive rehabilitation programs, with a focus on tailoring the treatment to the specific clinical needs of the patients.

***EM Hospital-based*** guides the process from patient selection to the definition of target objectives by the hospital team (Supplementary Figure 34; Figure 1).

**Figure 2:**
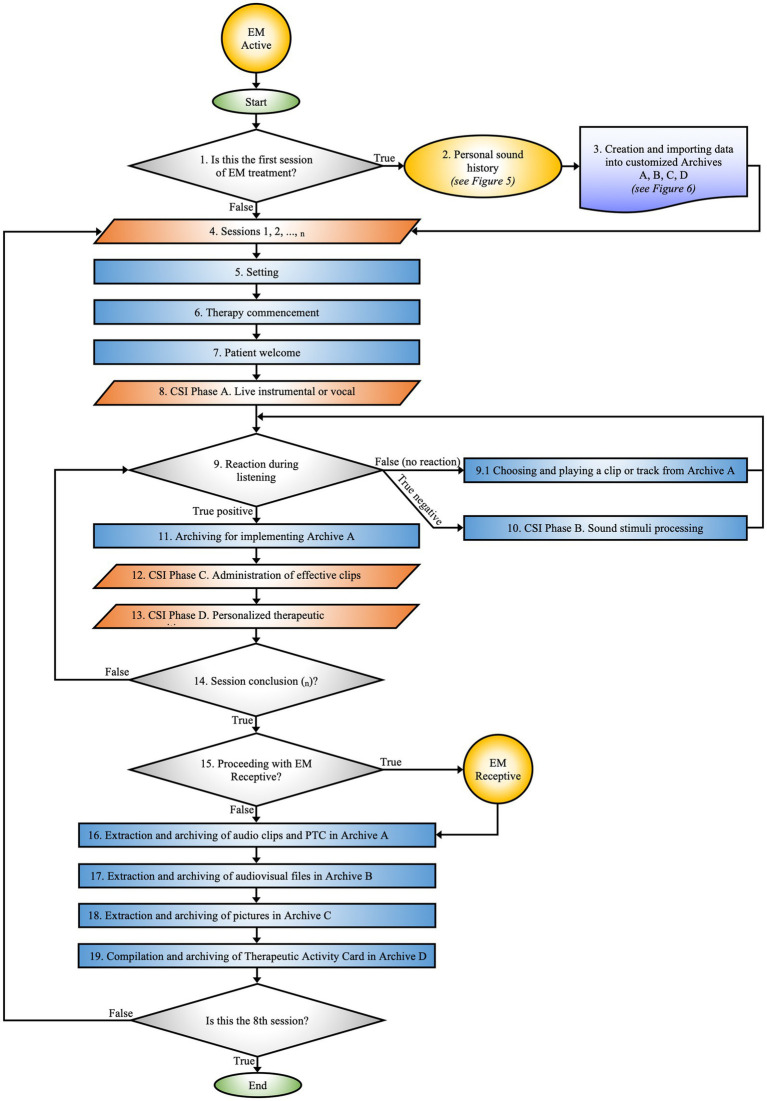
EM active. Maps the phases of the therapeutic process from the initial session to data archiving. The flow is guided by the patient’s responses to auditory stimuli.

***EM Active*** constitutes the core procedure of the EM, precisely delineating the *modus operandi* of MT activities (Figure 2).

**Figure 3:**
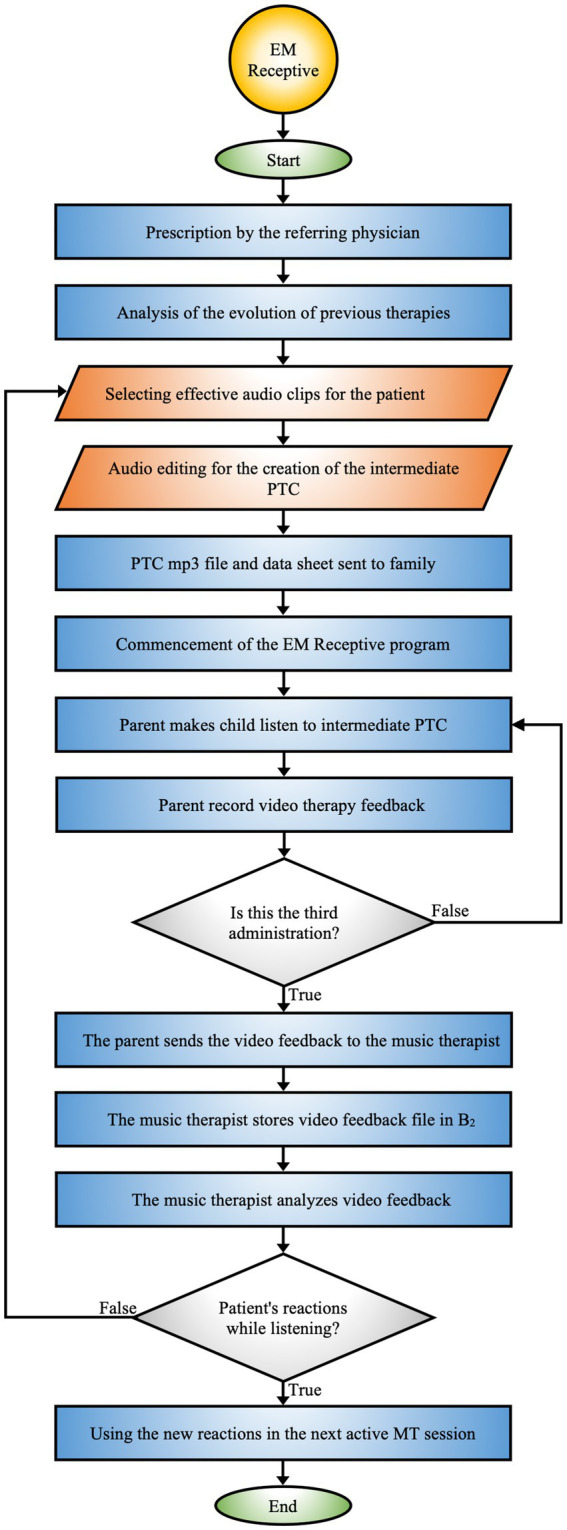
EM receptive. Protocol that integrates the collaboration between the hospital team, music therapist, and family to monitor and adapt the therapy based on the patient’s reactions.

***EM Receptive*** specifies the protocol for implementing rehabilitative activities on days when active MT sessions are not conducted in the hospital (Figure 3).

**Figure 4:**
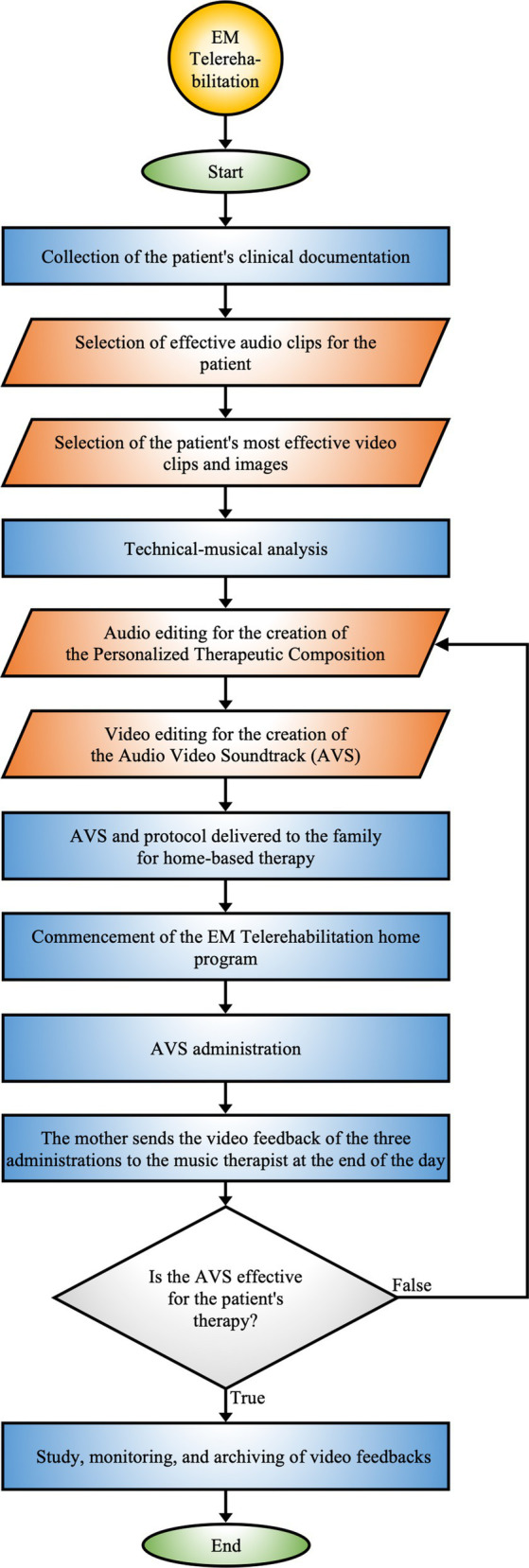
EM telerehabilitation. Process of home-based therapy utilizing personalized audio-video compositions, incorporating continuous monitoring and feedback to adapt the therapy according to patient responses.

***EM Telerehabilitation*** provides therapeutic continuity of MT directly in the patient’s home environment, offering the possibility of treatment delivery even in remote or difficult-to-access geographic settings. It can be prescribed by the hospital team or requested by parents, offering a structured pathway for remote therapy (Figure 4).

### 3.3 EM Hospital-based procedure

The **EM Hospital-based** protocol (see Figure 1) represents a rigorously defined methodological approach aimed at formulating specific clinical objectives for each patient. This protocol is designed to enable its application in various clinical contexts while ensuring interconnection with subsequent algorithms.

The selection of patients for the EM therapy program is carried out by a multidisciplinary team, including physicians, psychologists, head nurses, nurses, and certified music therapists, ensuring an integrated therapeutic approach. The prescription of the EM program is based on inclusion criteria, considering variables such as age, pathology, severity of the condition, and the optimal number of necessary sessions, allowing for precise treatment personalization.

After the patient selection process and before the start of the EM program, parents provide privacy consent for the inclusion of their children in the scientific studies and for video recording of the sessions. Furthermore, questionnaires are provided to the parents, who complete them independently. This allows for a preliminary assessment of both the patient and the parent (T0).

The entire sample is divided into two groups, experimental and control, through blind randomization. Subsequently, the physician and music therapist work closely to review the clinical history of the patients assigned to the experimental group to precisely define the therapeutic goals of the EM MT program. At the same time, the control group follows an intensive neurorehabilitation program without the EM MT intervention. Upon completion of the therapeutic path, which includes both intensive rehabilitation and MT sessions, T1 assessments are conducted for both groups to evaluate the effectiveness of the interventions and compare outcomes between the two groups.

Before discharge, if deemed necessary, the hospital team proposes that families from the experimental group continue the EM at home through the telerehabilitation program.

### 3.4 EM active procedure

The EM Active constitutes the detailed protocol for practical MT activities, designed according to the specific clinical needs and individualized treatment goals of each patient (Figure 2).

This process is carried out within the SR by a certified music therapist and a certified co-therapist. Activities may include learning exercises with musical instruments, improvisations, explorations of EmoTonePhonoSymbolisms (91), and sound elaborations, adapted to the patient’s needs and therapeutic progress. EM approach involves the administration of sensory stimuli using unimodal, multisensory, and cross-modal techniques, aimed at creating PTC.

#### 3.4.1 Is this the first EM session?

During the initial session, the music therapist informs the parent about the EM therapeutic path and outlines the objectives to be achieved during the rehabilitation intervention. The session comprises an observational assessment of the mother’s psychological state to encourage her active involvement in the therapeutic process and facilitate collaboration for subsequent stages.

#### 3.4.2 Personal sound history

**Figure 5:**
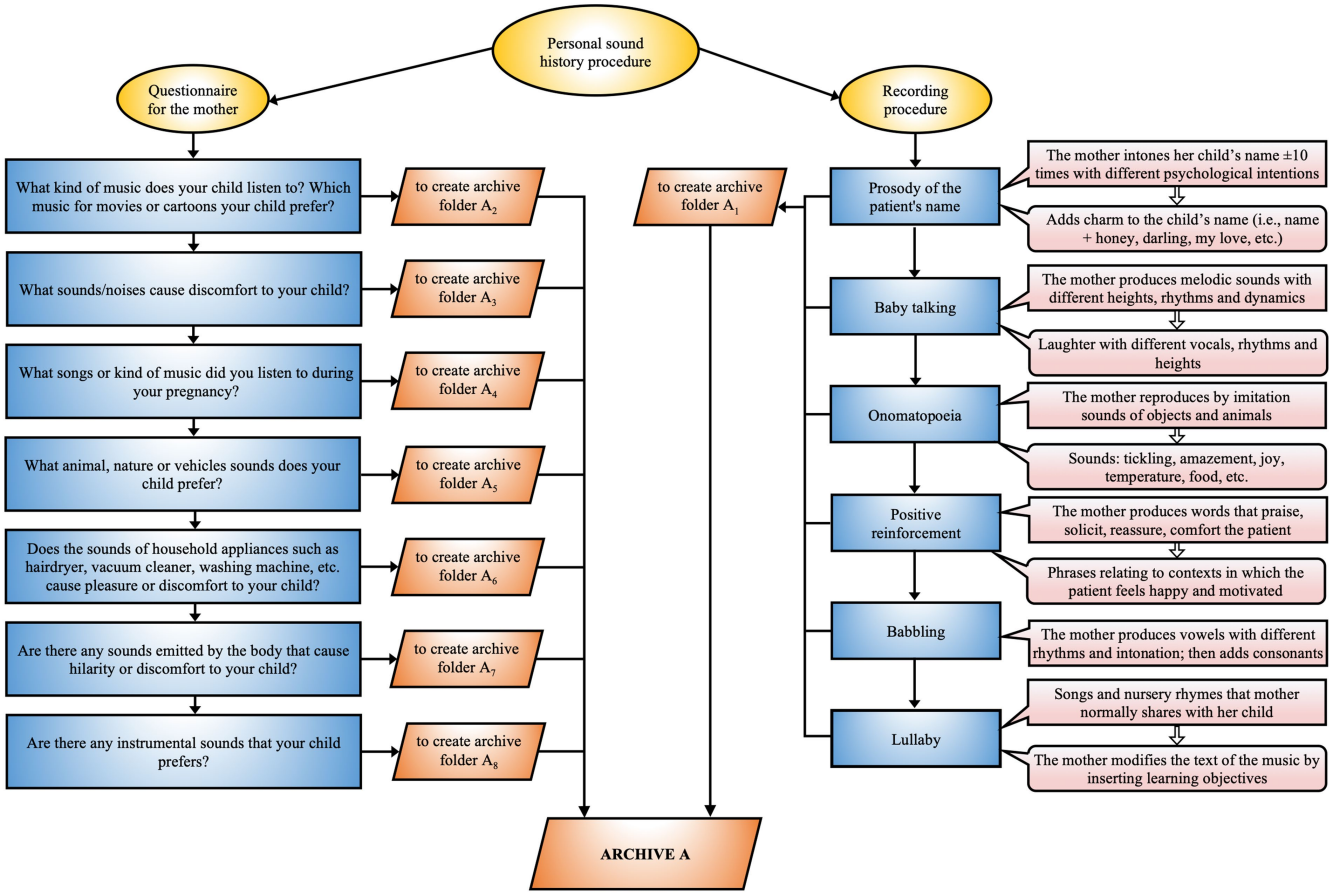
EM personal sound history. Procedure for acquiring the personalized sound history that integrates a questionnaire for the mother and a recording methodology aimed at identifying relevant auditory stimuli for optimizing the therapeutic process.

After reviewing the patient’s clinical history, the music therapist collects the “Personal Sound History” through the following procedure:

1. **Questionnaire**: A structured interview is employed to gather data on the patient’s sensory sensitivities, focusing on areas of vulnerability such as photosensitivity, auditory hyperreactivity, olfactory intolerances, tactile difficulties, and feeding issues. This information is essential for constructing a comprehensive sensory profile, which is indispensable for tailoring the therapeutic intervention (Figure 5).
2. **Mother’s voice recording**: The therapist records various linguistic elements of the mother, such as the prosody of the child’s name, baby talk, tenderness, positive reinforcement, and lullabies. The recording is done in a supportive environment to ensure that the mother feels comfortable and motivated.

#### 3.4.3 Creation and importing data into customized archives A, B, C, D

**Figure 6:**
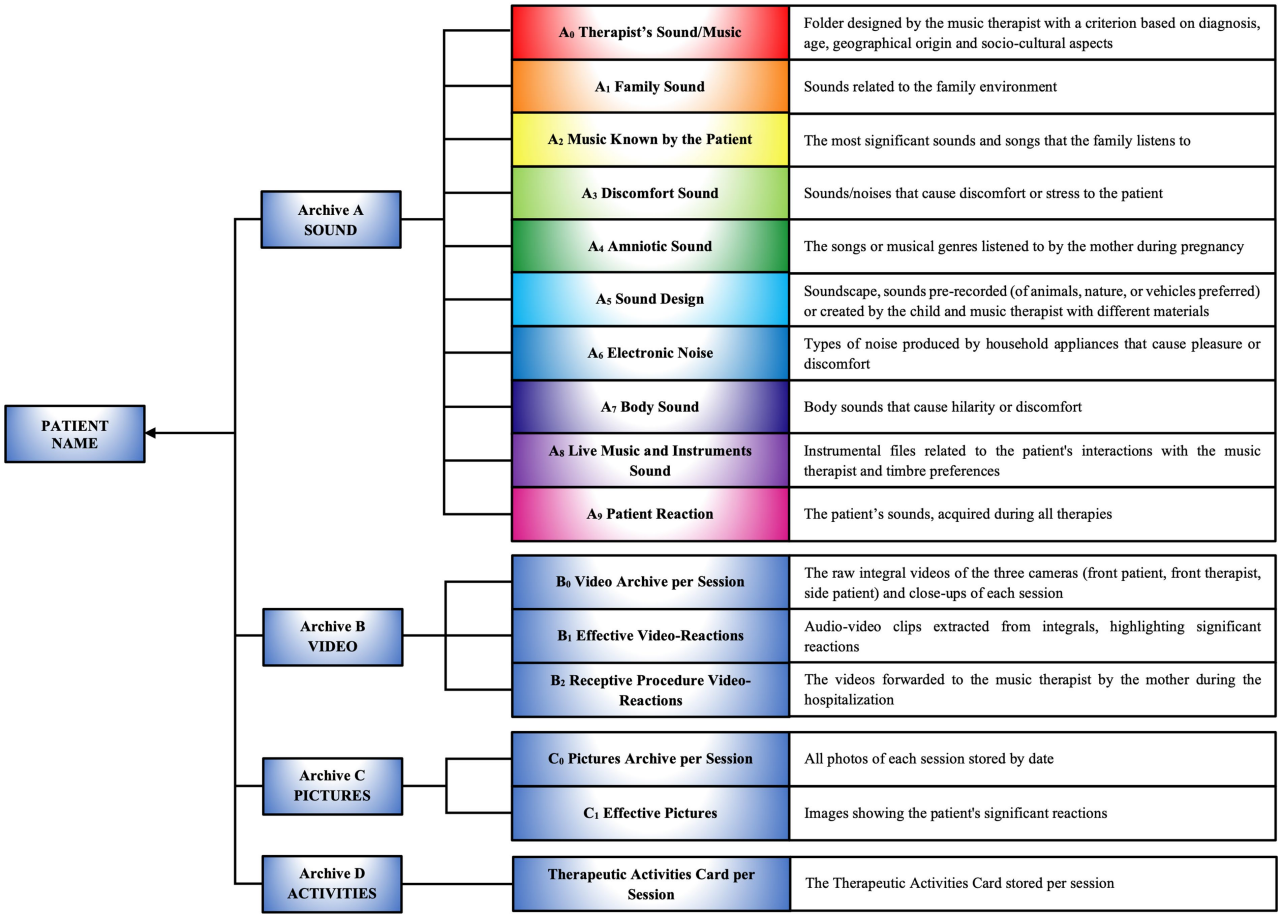
Creation and importing data into customized archives A, B, C, D. Structure of personalized archives containing data on the patient’s sounds, videos, images, and therapeutic activities, organized into categories to support the personalized therapeutic compositional process.

The music therapist analyzes the raw recording previously made with the patient’s mother, removing background noise and segmenting the audio files, which are then named according to their content. The selected files are used as auditory stimuli for therapy and as a notation of the compositional structure. The database, named after the patient, includes preferences derived from the questionnaire and is structured into four distinct archives (Figure 6; Supplementary 3.1):

1. Sound Archive A

- A_0_ Therapist’s Sound/Music
- A_1_ Family Sound
- A_2_ Music Known by the Patient
- A_3_ Discomfort Sound
- A_4_ Amniotic Sound
- A_5_ Sound Design
- A_6_ Electronic Noise
- A_7_ Body Sound
- A_8_ Live Music and Instruments Sound
- A_9_ Patient Reaction

1. Video Archive B
  - B_0_ Video Archive per Session
  - B_1_ Effective Video-Reactions
  - B_2_ Receptive Procedure Video-Reactions
2. Pictures Archive C
  - C_0_ Pictures Archive per Session
  - C_1_ Effective Pictures
3. Therapeutic Activities Archive D
  - Tables detailing the activities performed in each session (Supplementary Figure 14).

#### 3.4.4 Sessions 1, 2,.., n

All sessions are video recorded, an essential step to ensure an accurate evaluation of the patient’s responses and provide an in-depth retrospective analysis of the therapeutic process. This practice also facilitates rigorous documentation and optimizes the design of personalized musical compositions.

The program involves a minimum of eight sessions, conducted three times per week during the patient’s hospital stay. The duration of each session, typically ranging from 30 to 40 min, is adjusted based on the patient’s age and specific needs. Each session follows a therapeutic structure composed of sequential phases and steps, specifically tailored for each encounter.

#### 3.4.5 Setting

The sessions take place within the SR, a space designed to provide a highly flexible and resilient environment, capable of adapting to various age groups and specific psychophysiological conditions of the patients. This adaptability ensures maximum safety for both the patient and the parent during the therapeutic sessions.

The MT team is responsible for preparing the SR through a series of interventions:

- Cleaning and sanitizing of the surroundings, materials, and musical instruments
- Setting up the workstation
- Organizing sound material for playback, and balancing output signals from software and mixer
- Placement of aids for video recording, control of gain levels, and framing
- Connection and checking of LED lights and mobile LED heads, wired to the DMX interface
- Preparation of software for the touchscreen monitor (digital flipchart)
- Preparation of the projector for augmented reality
- Changing of hammock sheets
- Thorough handwashing and donning of brightly colored clothing by the therapist and co-therapist.

#### 3.4.6 Therapy commencement

During this phase, the music therapist and co-therapist define and coordinate their roles and responsibilities with respect to the patient and the therapy within the therapeutic environment.

#### 3.4.7 Patient welcome

##### 3.4.7.1 Step 1. Patient observation and feedback with the parent

The therapist assesses the psychophysiological condition of the patient and the parent before starting the therapy, using professional behaviors.

##### 3.4.7.2 Step 2. Patient introduction to the multisensory space

The therapist welcomes the patient and the parent, guiding them through the multisensory environment (SR), which is rich in colors, scents, sounds, and images, and encourages them to explore the space. Constant monitoring and guidance by the therapist are essential to ensure the child’s safety and facilitate a smooth transition into the environment.

##### 3.4.7.3 Step 3. Review of the previous session

Before beginning the MT session, the therapist reviews the activities of the previous session with the parent, checking for any changes in the child’s behavior and monitoring therapeutic progress. This feedback process ensures that the therapeutic plan remains appropriate to the patient’s psychophysiological needs and promotes parental involvement in achieving clinical objectives.

#### 3.4.8 Compositional sound interventions—phase A. Live instrumental or vocal

CSI are structured therapeutic approaches that employ personalized musical and auditory techniques to achieve specific clinical objectives. The CSI process is divided into four phases:

- Live Instrumental or Vocal
- Sound Stimuli Processing
- Administration of Effective Clips
- Personalized Therapeutic Composition (PTC).

*Phase A. Live Instrumental or Vocal* has as its main objective the accurate monitoring of the patient’s responses to assess his emotional and psychophysiological state. The therapist observes and documents every verbal and non-verbal response of the patient to establish a clinical baseline, which is essential for early detection of signs of discomfort or specific needs that may require targeted interventions. Therapy begins with silence, conceived as an active element (54), intended to facilitate and promote the patient’s adaptation within the therapeutic space.

This silence fosters concentrated attention and a gradual anticipation of auditory intervention, allowing the music therapist to establish initial non-invasive contact in a secure and trusting environment. Subsequently, the therapist uses the sound of their own breath, alternated with silence, to synchronize with the patient’s breathing cycle, generating interest and attention. The music therapist introduces hand-clapping sounds, synchronized with the patient’s breathing, and uses a sound level meter to identify the auditory threshold. At this stage, soft and warm vocal sounds, imitating the mother’s voice, are emitted at an intensity of 30–35 dB. These sounds are used to initiate active interaction and modulate emotions in synchrony with the patient’s heart rate and breathing variations (92–94).

Next, the real-time use of a musical instrument gradually introduces auditory stimuli with varying intensities, including consonant and dissonant intervals, glissandi, harmonics, and fragments of familiar songs or cartoon themes (95–98). The administration of such auditory stimuli often reveals significantly stronger responses in many patients when the clarinet is used. These patients exhibit a keen interest in following the sound trajectory in sync with the movement of the instrument, suggesting a deeper sensory and cognitive engagement in musical perception.

Additionally, it is noteworthy that the alternation between a familiar melody and intervals of silence induces predictive anticipation of the melody’s return in patients, suggesting an active expectation that predisposes them to emotional responses.

When the therapist introduces unfamiliar sounds, such as harmonics perceived as whistles, motor excitement and emotional surprise are observed, highlighting an innate curiosity toward new auditory stimuli. With controlled and repeated exposure to these sounds, patients develop progressive adaptation, demonstrating increased tolerance and predictive capacity, accompanied by moments of laughter. This process suggests sensory and cognitive adaptation, where acquired familiarity with unfamiliar sounds not only reduces initial surprise but also enriches the patients’ emotional and social experience.

Supporting this, many studies highlight that the temporal dynamics of auditory signals contain significant information (99–103). The brain is thought to actively anticipate forthcoming auditory stimuli in consideration of prior contextual information; this process is known as predictive coding. This mechanism, essential for learning and language comprehension, as well as for musical processing of pitch and rhythm, has been extensively documented by various authors in audiology (104–111).

#### 3.4.9 Reactions during listening

In this step, the music therapist manages and controls the Digital Audio Workstation to synchronize and adapt sounds in real-time to the activities carried out by the co-therapist with the patient. This setup facilitates real-time musical composition and effective synchronization of therapeutic activities. The primary goal is to observe, evaluate, and understand the patient’s reactions to novel auditory stimuli.

##### 3.4.9.1 Choosing and playing a clip or track from archive a

If the patient does not exhibit any response during exposure to the audio clips, several possibilities should be considered:

- The musical stimulus may be inadequate for the patient’s specific needs
- The patient’s emotional or physical state may be influencing their responsiveness
- The patient may be disengaged or distracted
- A lack of response could suggest the need for further diagnostic evaluations for a more in-depth analysis.

#### 3.4.10 CSI phase B. Sound stimuli processing

During exposure to new auditory stimuli, it is essential to adopt a sensitive therapeutic approach, particularly if the stimuli elicit negative reactions. In such cases, it is necessary to immediately cease sound administration and monitor the patient’s responses, which may manifest through facial expressions, body movements, vocal tone variations, or other signs of discomfort. If the negative stimulus stems from sounds or words essential to the patient, such as the mother’s voice or natural sounds (soundscape), it is fundamental to process and adapt the sounds using appropriate techniques until acceptance is achieved. This issue is especially pertinent for long-term hospitalized patients who may experience alterations in sensory or relational learning (112).

Sound processing and modification techniques include amplitude adjustment, compression, delay, echo, and pitch correction. Key parameters for developing new real-time auditory stimuli that address the emotional and psychological needs of the patient include intensity, spatiality, directionality, speed, and frequency (Supplementary 3.4).

The therapist employs two playback methods to assess the efficacy of the sound stimuli: one involves the sequential presentation of processed musical clips, while the other overlays effective clips with unknown sounds.

The goal is to facilitate the acceptance of unfamiliar sounds, such as traffic and thunder, to improve quality of life and develop skills in managing environmental sound stimuli.

#### 3.4.11 Archiving for implementing archive A

The music therapist extracts the patient’s positive reactions from the raw audio recordings of the ongoing therapy. The acquired clips are timestamped, named according to content, and archived in the A_9_ Patient Reactions folder. The process ensures that the therapist has immediate access to new sound material for use in the subsequent step of therapy.

#### 3.4.12 CSI phase C. Administration of effective clips

**Figure 7:**
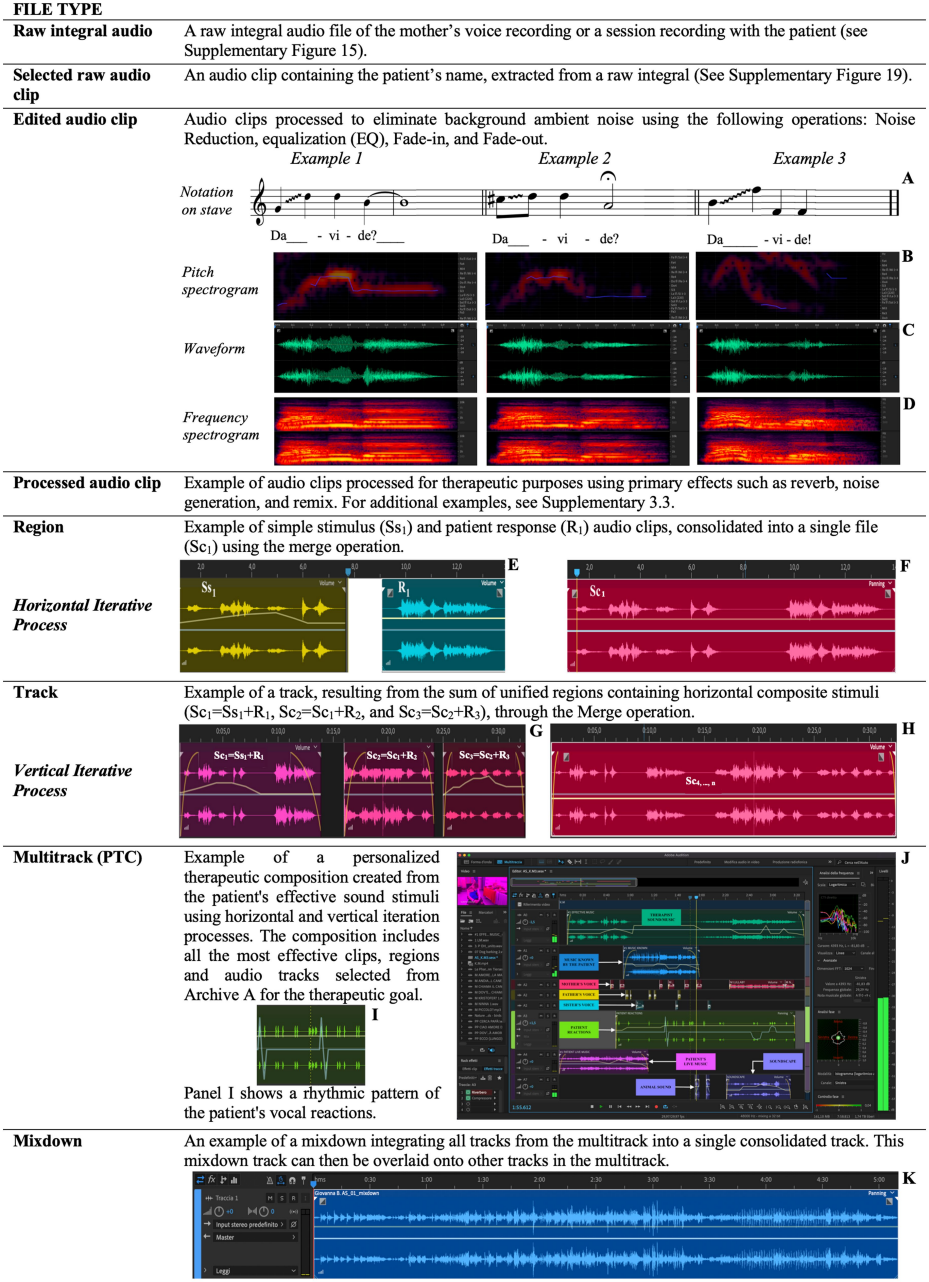
This figure explicates the technical and compositional steps involved in creating and managing sound stimuli, integrating patient responses, and progressively developing a therapeutic pathway through iterative processing and personalized composition. Examples of vocal clip notations on the musical stave (A) alongside their pitch spectrograms (B), waveforms (C), frequency spectrograms (D). Consolidation of two sound clips, i.e., Ss_1_ and R_1_ (E), into a single new file (F). Consolidation of the Sc_1_, Sc_2_ and Sc_3_ clips (G) in the track (H). A rhythmic pattern focused of the patient’s vocal reactions (I). Illustration of the PTC (Personalized Therapeutic Composition) processing within the software’s multitrack interface (J). The track produced from the Mixdown process, showcasing the iterative and personalized approach to therapy through sound integration (K).

In this phase, a sequence of auditory stimuli organized in *rhythmic patterns*, based on the patient’s vocal reactions, is presented. The rhythmic patterns, designed as cyclic structures, regulate the temporal flow of music through the use of elements such as tempo, accents, rhythmic subdivisions, and meter (Figure 7I).

In the therapeutic context, these patterns serve as temporal organizers, facilitating the emergence and integration of the patient’s vocal responses into repeatable models. Reproducing the patient’s vocal reactions, structured within these patterns, enables the music therapist to verify the patient’s ability to recognize their own voice, analyze the effects produced, and assess their skill in following the trajectory of the sound source.

This procedure acts as a tool for evaluating therapeutic efficacy and supports the patient’s neurocognitive adaptation, promoting improvements in their ability to consciously recognize and reproduce their own voice.

In the following, new auditory stimuli specifically associated with congruent images are presented. An example of such correspondence would be the sound of a dog barking, synchronized with the display of an image depicting a dog. This technique aims to establish and enhance the synergy between visual and auditory processing, carefully modulating the relationship between image size and perceived sound intensity. Variables such as the distance of the sound source from the patient, the speed of sound approach or withdrawal, and the direction from which it originates are also considered. These factors are orchestrated to strengthen multimodal sensory connections, with the goal of fostering optimal sensory integration and a more coherent adaptive response from the patient, both cognitively and perceptually. This assessment allows for the adaptation and modification of subsequent clips to optimize the therapy.

The treatment proceeds with the sequential or overlapping reproduction of clips to create a more complex, realistic, and engaging auditory experience that reflects the relationships between sound and environment.

#### 3.4.13 CSI phase D. Personalized therapeutic composition

In the final phase of CSI, the music therapist integrates all positive effects observed in previous phases to create a PTC with logical continuity. The assessment of the patient’s emotional, cognitive, and behavioral responses guides the therapist in the selection and organization of musical and textual elements, aligning with therapeutic goals.

The “perceptual hierarchical function” within the compositional structure of the PTC is configured as a mechanism for organizing and prioritizing sound materials based on the patient’s positive responses. This process involves creating and managing a progressive sequence of compositional levels, which are continuously optimized and grouped according to their therapeutic efficacy. The hierarchical arrangement of sound elements, which include *sound cells*, clips, or compositional fragments such as individual voices (e.g., that of the patient, family members, or cartoon characters), is determined through a systematic analysis of the patient’s recognition and responsiveness to such stimuli. This approach allows the music therapist to specify the order of appearance, progression, and overlapping of clips within the PTC, thereby optimizing therapeutic responses and achieving specific clinical objectives.

**Figure 8:**
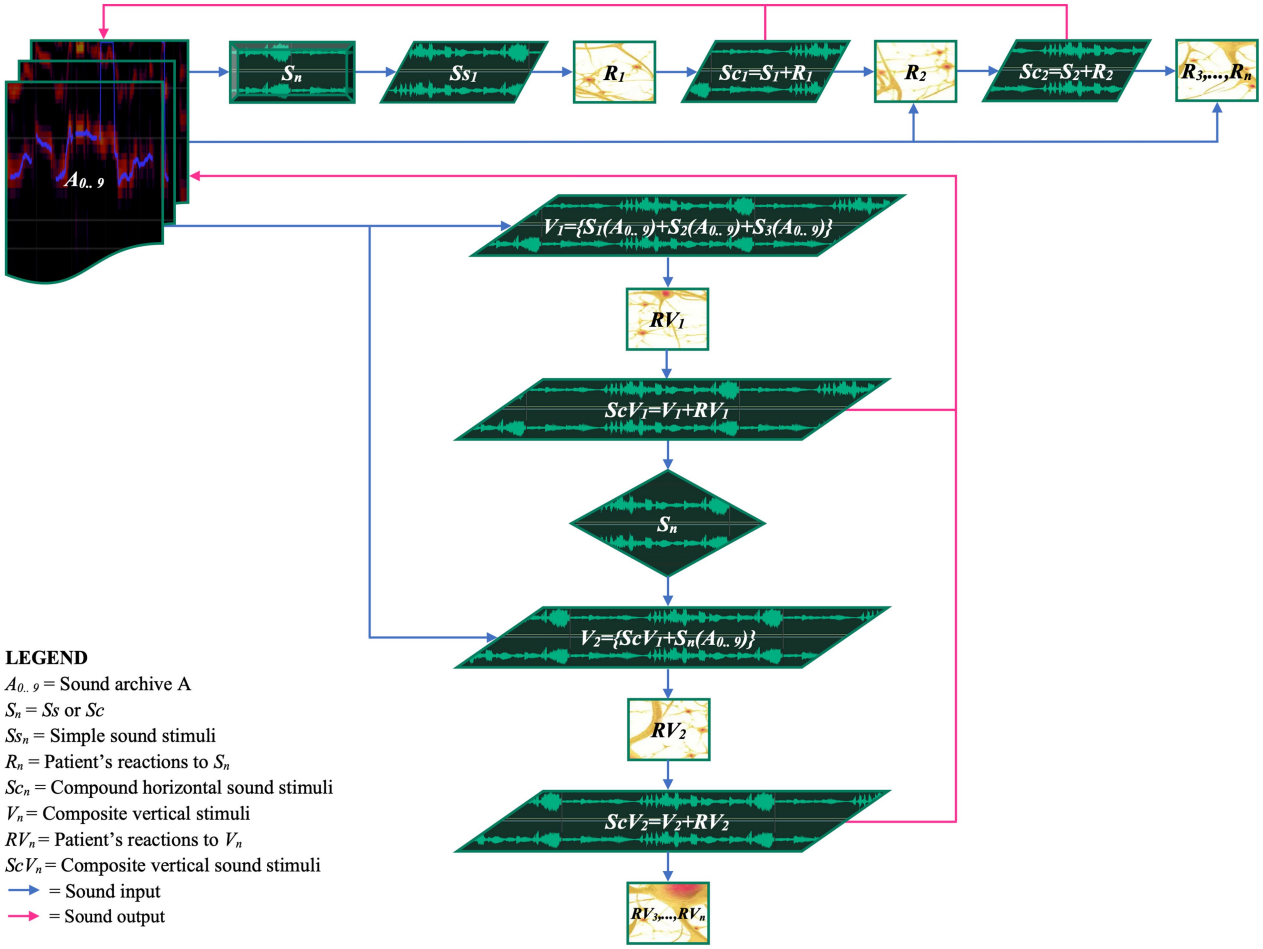
EM horizontal and vertical iterative schema of stimuli and patient’s responses. The figure shows the iterative process designed to create a dynamic and personalized therapeutic pathway based on the continuous reactions of the patient. Sound archives (A_0_..A_9_) are utilized to administer sound stimuli (S_n_) to the patient. The physiological and behavioral reactions of the patient (R_n_, RV_n_) are recorded and reintegrated into the therapeutic process, with the aim of dynamically adapting the stimuli. The mathematical expressions visualize how stimuli and responses interact within the iterative cycles, promoting a continuous personalization of the therapy.

To represent and synthesize the interaction between auditory stimuli and patient responses, we have developed an iterative schema (Supplementary Figure 35; Figure 8; Supplement 3.5). This schema shows how an auditory stimulus and the patient’s response merge to generate subsequent articulated stimuli. The goal is to create original and personalized musical sonorities, segments, and phrases based on the patient’s positive reactions. The iterative process can be implemented in two modes: sequential (melodically) and simultaneously superimposed (polyphonically).

##### Horizontal iterative process (melodic)

In each horizontal iteration, an auditory stimulus (*Ss_1_*) is presented to the patient. The patient’s response (*R_1_*) is fused with the original stimulus, resulting in a compound stimulus (*Sc_1_*; Figures 7E,F). This compound stimulus is archived and becomes the input for the next iteration.

##### Vertical iterative process (polyphonic)

The vertical iterative process is analogous to the creation of a polyphony, forming a complex set of stimuli to which the patient is invited to respond. The realization develops through overlapping stimuli administered simultaneously, such as *{S_1_(A_0_.. _9_) + S_2_(A_0_.. _9_) + S_3_(A_0_.. _9_)}*, fused with the patient’s response (*RV_1_*) to generate a new composite stimulus (*ScV_1_*). This compositional technique is relevant as the patient’s response not only reveals reactions to the stimuli but also provides valuable insights into the understanding and acceptance of the new auditory material presented (Figures 7G,H).

The compositional process continues through the use of harmony based on tonal intervals corresponding to the maternal voice spectrum. An example might be the use of a suspended cadence, where chords such as I-V or IV-II-V create a sense of questioning, perceived as expectation and tension. To reinforce and maintain this perception, a question pronounced by the mother’s voice is inserted into the multitrack, superimposed on the suspended dominant chord. This arrangement serves to amplify the anticipatory tension inherent in the harmonic structure, thus engaging the patient’s auditory and emotional process at a deeper level.

Subsequently, a silence interval is introduced to stimulate the patient’s response. The sequence continues with a harmonic resolution, in which the dominant chord (V) resolves naturally over the tonic chord (I), creating a sense of predictability, relaxation, and conclusion. This harmonic resolution is further enriched through the integration of the patient’s previous vocal reaction within the tonic chord, thus enhancing the effect of recognition and emotional gratification.

This compositional process aims to generate original sound material to be integrated into the PTC. The provided example shows one of many musical techniques designed to stimulate positive therapeutic responses, based on the patient’s musical culture. It is essential to incorporate melodies and harmonies characteristic of the patient’s musical tradition to maximize the effectiveness of the PTC.

Numerous studies demonstrate the effect of music on tone perception and sound environment, supporting iterative and compositional processes. In particular, tonal organization and its perception are central to the musical process (113). This concept is based on a tonal hierarchical structure that facilitates perception, memory, and musical execution, generating expectations in the listener (114).

Research conducted by Shepard and Jordan and Tillmann et al. provide empirical evidence demonstrating that listeners automatically use this tonal knowledge while listening (115, 116).

At the end of this phase, it can be confidently asserted that the sound material produced during sessions and all categorized sounds offer a wide range of possibilities for creating infinite PTCs.

##### Mechanisms of action of personalized therapeutic compositions

PTCs, based on personalized sound and musical stimuli, not only facilitate the interaction between the patient and parent but also exert a direct influence on numerous neurophysiological and psychological processes.

In the structure of the PTC, the perception of the maternal voice represents a fundamental component of infant development, influencing various aspects of the child’s behavior and cognitive and emotional development (117–120). Elements such as the prosody of the child’s name, positive reinforcement, and “motherese” are recognized for their positive impact on mood, motivation, and communication in therapeutic contexts (121–124). Previous studies have highlighted that listening to the recorded maternal voice elicits a complex response in the child, characterized by strong motor interest, contextual cognitive processing, and positive emotional response (36, 125, 126).

In motor terms, exposure to the maternal voice stimulates the child to orient toward the sound source, demonstrating active interest in tracking the sound trajectory. This motor response suggests particular attention and sensitivity to the mother’s voice, indicating an important foundation for the development of communicative and social skills.

From a cognitive standpoint, listening to specific words pronounced by the maternal voice within the musical structure of the PTC facilitates contextualization, meaning attribution, and content understanding for the patient. This phenomenon occurs through the combination of elements such as overlay of the maternal voice modulated to major chords, precise rhythmic patterns, evocative soundscapes of past experiences, and effective melody fragments. Such combination aids the child in navigating the sound space, facilitates memorization of auditory information, and allows understanding of emotional meaning contextualized within the surrounding environment. Furthermore, PTC combines sound stimuli with the patient’s responses, contributing to the consolidation of neural pathways through an associative learning process. Regular repetitions within the PTC allow the brain to recognize and memorize sound patterns, thereby strengthening both short-term and long-term memory (127, 128).

From the emotive point of view, interaction with the maternal voice during the PTC induces a range of positive emotional responses in the child, including joy, empathy, and the desire to be embraced and supported. This emotional response indicates the quality and nature of mother–child interaction, highlighting the importance of affective bond in promoting emotional and social development of the child.

The mechanisms of action and effectiveness of the administration of PTCs could be traced back to the effects of music on the brain. Different studies provide evidence that MT has beneficial effects on functional brain activity and connectivity in networks underlying higher-order cognitive, socio-emotional, and motor functions. In fact, music perception can activate various limbic and paralimbic structures and improve network connectivity in children and adults (63, 64, 129). Music modulates synaptic plasticity and promotes neurobiological processes, neuronal learning, and readjustment in the human and animal brain (130, 131).

#### 3.4.14 Session conclusion (_n_)

The conclusion of the therapeutic session is an important step for consolidating the work done and preparing both the patient and the parent for the continuation of the therapeutic path. This step is divided into three phases.

##### 3.4.14.1 Step 1. Gradual inform the patient of session conclusion

The therapist signals to the patient, either verbally or non-verbally, the approach of the session’s end, ensuring a gradual closure. This allows the patient to mentally prepare for the conclusion and to complete any communicative and emotional-relational needs.

##### 3.4.14.2 Step 2. Sharing emerging changes with the parent

The therapist updates the parent on the changes or progress observed during the session, actively involving them and reinforcing the collaboration between the therapist and the family.

##### 3.4.14.3 Step 3. Personalized “greetings” with the patient

The therapist concludes with personalized goodbyes, thanking the patient for their engagement and encouraging them for the work accomplished. This helps maintain a positive and motivating therapeutic environment.

#### 3.4.15 Proceeding with EM receptive music therapy?

The receptive procedure, proposed by the rehabilitation team for pediatric patients with prolonged hospital stays, aims to compensate for the limited sensory and auditory exposure to the external world, thereby facilitating multisensory integration and supporting cognitive and emotional recovery.

The procedure is reported and detailed in Section 3.5 of the text and in Figure 3.

#### 3.4.16 Extraction and archiving of audio clips and PTC in archive A

At the conclusion of the therapeutic session, the music therapist extracts audio tracks from the devices used (Supplementary 3.2). The most relevant clips are selected and archived in Archive A for future reference. Additionally, the intermediate PTC is archived for use in subsequent sessions.

#### 3.4.17 Extraction and archiving of audiovisual files in archive B

The therapist extracts and catalogs raw session material into the folder “B_0_ Video Archive per Session,” organizing it by date. The footage is then reviewed, edited into shorter clips, and archived in the folder “B_1_ Effective Video-Reactions” to monitor significant changes in patient behavior. Additionally, video feedback from patients completing EM Receptive or EM Telerehabilitation procedures is chronologically archived in the folder “B_2_ Receptive Procedure Video-Reactions.”

#### 3.4.18 Extraction and archiving of pictures in archive C

Images extracted from session videos are stored in the folder “C_0_ Picture Archive per Session.” The most relevant photos, selected based on session activities, therapeutic goals, and emotional states, are transferred to the folder “C_1_ Effective Pictures” to document clinical progress and new learnings.

This process enables the creation of the Audiovisual Soundtrack (AVS) for home therapy protocols, as indicated by Bompard et al. (35).

#### 3.4.19 Compilation and archiving of therapeutic activity card in archive D

This document summarizes the activities performed during each session, the materials used, relevant musical bibliography, the tonalities employed, and the patient’s responses. It provides a useful overview for a detailed evaluation of therapeutic practices and patient responses, aiding in the adaptation and refinement of therapeutic strategies (Supplementary Figure 14).

### 3.5 EM receptive procedure

The EM Receptive intervention, prescribed by the hospital team, is implemented during the hospitalization, targeting pediatric patients in early developmental stages or those undergoing prolonged hospital stays. These children often experience limited exposure to sensory and auditory stimuli, which can potentially hinder their neurocognitive development (112). The intervention aims to compensate for this lack by optimizing well-being through auditory stimulation tailored to the therapeutic goal.

The EM Receptive procedure unfolds in a series of phases, grounded in a continuous monitoring model and dynamic adjustment (Figure 3).

#### 3.5.1 Initiation of the EM receptive program

The therapeutic path begins with a retrospective analysis of the patient’s previous sessions during hospitalization, aimed at identifying sensory gaps, deficits, and issues. This initial evaluation allows for the customization of compositional sound interventions, thereby optimizing learning and enhancing receptivity to maximize the rehabilitative process’s effectiveness.

#### 3.5.2 PTC creation

Based on the information gathered, the sound clips most relevant to the patient are selected. A PTC is then created, designed to stimulate emotional and cognitive responses by the use of soundscapes and everyday auditory stimuli. Audio materials are chosen according to the patient’s “*perceptual hierarchical function*” and enriched with novel and unfamiliar sounds essential for the patient’s life.

The final file, exported in MP3 format and accompanied by a technical sheet containing detailed instructions for administration, is provided to the family. The parent assumes responsibility for managing the PTC following the provided guidelines.

#### 3.5.3 PTC administration

The PTC is administered via a Bluetooth speaker, operated by the parent, with sound intensity regulated between 40 and 50 dB. Therapeutic sessions take place in the afternoons without active MT, during which the PTC is proposed three times at two-hour intervals. This schedule not only promotes the assimilation of auditory experiences but also ensures adequate recovery between exposures. These intervals allow for careful monitoring of the patient’s emotional and cognitive responses, facilitating therapeutic adjustments when necessary. The PTC has a length of approximately 2–3 min, a time designed to maintain the child’s attention and optimize therapeutic outcomes, while avoiding sensory overload. During the administration, the parent video records the child’s reactions.

#### 3.5.4 Feedback collection and transmission

The parent sends the video feedback to the music therapist, who archives the material in the patient’s B_2_ folder. This system consents a systematic monitoring of the child’s responses to the musical intervention and facilitates a comprehensive evaluation of the PTC’s effectiveness.

#### 3.5.5 Feedback analysis and adaptation

The music therapist reviews the video feedback, focusing on the patient’s sensory and behavioral responses during listening sessions of the PTC. This analysis enables the evaluation of the impact of the proposed sounds and allows for targeted modifications to the musical composition based on the observed reactions.

These adjustments stimulate neural circuits related to auditory processing and emotional regulation, promoting the reorganization of neural networks (132). This process optimizes the integration of auditory experiences, enhancing the patient’s responsiveness. Continuous feedback enables the therapist to further refine the intervention, ensuring a progressively more personalized and effective therapeutic approach.

#### 3.5.6 Interaction and integration with EM active

Information collected through the EM Receptive procedure is utilized to improve active MT sessions. The reactions observed during receptive administrations provide the therapist with insights to refine the EM Active intervention, maximizing the overall therapeutic efficacy.

### 3.6 EM telerehabilitation procedure

EM Telerehabilitation, prescribed by the hospital team or requested by parents, enables discharged patients to continue MT at home (Figure 4).

The goal of EM Telerehabilitation is to ensure the continuity of the rehabilitation process within the home environment. This therapy not only preserves the progress obtained but also stimulates further improvements, adapting to the patient’s developmental needs and home context. Additionally, the importance of telerehabilitation lies in its ability to reach areas where rehabilitation facilities are limited or absent, thus providing treatment opportunities for patients in remote locations (133).

The music therapist initiates the EM Telerehabilitation process by reviewing the patient’s clinical documentation, which is fundamental for customizing the intervention.

The technical process begins with a detailed analysis of the patient’s database, which includes the personalized archives generated during hospitalization and EM MT activities. In this initial phase, recorded audio-video clips are evaluated, focusing on patient reactions, particularly reaction times and attention levels. Detailed analyses of musical bibliography and personalized music are conducted to identify those that have demonstrated the greatest therapeutic efficacy.

From this data, a detailed graph is created to provide an overview of the MT path made (Supplementary 4).

Next, a PTC is composed according to the specific treatment goals. In addition, significant videos and images documenting key moments of the patient, such as smiles, interactions, and activities stimulated by MT interventions during hospitalization, are extracted from Archives B and C. These elements are then overlaid onto the PTC using video editing software to create an Audiovisual Soundtrack (AVS). The technical criterion for overlaying images and videos is based on the alignment of significant moments experienced by the patient (e.g., expressions of joy and participation) and the PTC music. This approach allows for synchronizing the patient’s emotional and behavioral reactions with the PTC’s acoustic features.

The duration of AVS generally ranges from 3 to 4 min and is tailored to the patient’s specific treatment goals.

Once the AVS is completed, the music therapist instructs the family on administration procedure using a technical sheet that details timing and methods.

The AVS should be administered using an appropriate device, preferably a television, for 12 consecutive days. The protocol involves three daily sessions, with the final session scheduled at least 3 h before bedtime. A 24-h break is also included on the seventh day. Sessions, scheduled from early afternoon and spaced at least 2 h apart, accommodate family commitments.

The parent administers the treatment to the child, involving, when possible, the entire family in active MT activities.

During AVS administration, the parent documents the patient’s responses by sending video feedback to the music therapist for continuous monitoring and potential technical adjustments. These files are archived by the therapist to monitor treatment evolution and adapt the intervention to the patient’s emerging needs.

The AVS, combining images and videos with the PTC, creates a personalized short film where the patient is the protagonist. This approach enhances therapeutic efficacy, significantly improving the patient’s emotional and motivational engagement.

EM Telerehabilitation has shown statistically significant results in sleep disorders and reducing parental stress (35).

### 3.7 Participants

The study by Bompard et al. included 20 children with developmental delay who had participated in the EM program prior to the pandemic. Of these, 12 completed all home sessions and assessments and were therefore eligible for data analysis (35).

The subsequent research, by Liuzzi et al., selected 58 children from a larger cohort, randomized into an experimental group (*n* = 35) and a control group (*n* = 23); among these, 25 children in the experimental group and 10 in the control group completed assessments at both T0 and T1 (36).

The procedures were conducted in accordance with the Declaration of Helsinki, with informed consent obtained from the parents.

### 3.8 Measures

The measures adopted in the study by Bompard et al. (35) included the Sleep Disturbance Scale for Children (SDSC), used to assess sleep quality (134), and the Parenting Stress Index - Short Form (PSI-SF), employed to analyze parental stress (135). In the subsequent research by Liuzzi et al. (36), additional assessments were introduced, including the Italian Questionnaires of Temperament (QUIT), which investigates the child’s behavior in various social and play contexts (136), and the Pediatric Quality of Life, which explores the quality of life of children through age-specific modules, also including the Family Impact Module intended for parents (137–139).

### 3.9 Statistical analysis

The data from the first feasibility study of EM were analyzed using MedCalc software, version 12.7 (MedCalc Software, Belgium). Categorical variables were reported as absolute numbers and percentages, while continuous variables were expressed as median and standard deviation if normally distributed, assessed using the D’Agostino–Pearson test. Differences in pre-and post-treatment measures were examined using the paired t-test. A two-tailed *p*-value <0.05 was considered statistically significant (35).

For the second study, statistical analyses were conducted using STATA software, version 17 (StataCorp LP, College Station, TX). Data normality was assessed using the Shapiro–Wilk test. Categorical variables were summarized in absolute frequencies and percentages, while continuous variables were expressed as mean and standard deviation. To determine statistical differences in scores between baseline (T0) and post-treatment (T1), the paired t-test was used for continuous variables. Additionally, scores between subjects in the experimental group and the control group were compared at both baseline and post-MT using the independent t-test, while categorical variables were compared using the Chi-square test or Fisher’s exact test. Statistical significance was set at *p* < 0.05 (36).

## 4 Results

The application of EM resulted in significant improvements across various key areas for children with neurodevelopmental disorders. Previous studies have provided relevant statistical evidence regarding the positive effect of EM on sleep quality, emotional regulation, and the reduction of parental stress (35, 36). We reiterate previous data to support the analysis of the results obtained, thereby consolidating the evidence of EM’s effectiveness. This integration strengthens the evaluation of the observed improvements in key areas, providing a solid comparative basis for the current study.

### 4.1 Children

In the study by Bompard et al. (35), a statistically significant improvement in sleep quality was recorded. The total score on the SDSC decreased from 49.5 ± 19.7 to 40.5 ± 10.5 (*p* = 0.010), indicating an overall improvement. Specifically, sleep breathing disorders dropped from 6.5 ± 3.6 to 4.4 ± 2.7 (*p* = 0.012), and sleep–wake transition disorders improved from 10.6 ± 4.7 to 8.8 ± 2.8 (*p* = 0.032). These findings were confirmed by Liuzzi et al. (36), who observed a statistically significant reduction in the total score on the SDSC from 42.4 ± 14.5 to 37.7 ± 12.7 (*p* = 0.031). Additionally, disorders related to the initiation and maintenance of sleep decreased from 14.6 ± 7.1 to 13.4 ± 6.1 (*p* = 0.050), while sleep–wake transition disorders further improved from 9.6 ± 3.3 to 8.0 ± 2.9 (*p* = 0.026).

Furthermore, concerning emotional control, Liuzzi et al. (36) noted a statistically significant increase in the positive emotionality score of the QUIT, rising from 4.1 ± 1.4 to 4.4 ± 1.2 (*p* = 0.013), demonstrating the effectiveness of EM in emotional regulation.

### 4.2 Parents

Bompard et al. (35) show a statistically significant reduction in parental stress. The PSI-SF score related to parental distress decreased from 27.4 ± 10.1 to 24.4 ± 10.2 (*p* = 0.001), accompanied by an improvement in defensive responding, which fell from 16.8 ± 5.9 to 14.8 ± 6.3 (*p* = 0.012).

Finally, the study by Liuzzi et al. (36) reported statistically significant improvements in family quality of life as measured by the Pediatric Quality of Life, with the emotional functioning score increasing from 61.6 ± 26.4 to 69.6 ± 22.6 (*p* = 0.029), and social functioning improving from 61.5 ± 25.4 to 68.3 ± 26.4 (*p* = 0.012). Statistically significant improvements were also recorded in worry (*p* = 0.032), daily activities (*p* = 0.032), total score (*p* = 0.039), and health-related quality of life (*p* = 0.035), highlighting the positive impact of EM on the entire family.

## 5 Critical analysis

### 5.1 Ethical considerations in integrating music technology with neuroscience

The integration of music technology with neuroscience in therapeutic contexts raises significant ethical issues. A fundamental aspect concerns informed consent, which is particularly important when working with vulnerable populations, such as children.

It is essential for therapists to ensure that patients and their caregivers have a full understanding of the interventions, the use of technology, and any potential risks involved (140). This transparency is essential for maintaining trust and guaranteeing the autonomy of participants.

Moreover, privacy protection and data security are imperative when using technologies that collect sensitive patient information. Such data must be anonymized and securely stored in accordance with the General Data Protection Regulation (141), to ensure patient confidentiality.

Lastly, there is a risk of biases and inequities in access to music technology. Therapists need to be aware of cultural, socioeconomic, and individual differences that may influence how patients respond to music technology. Ensuring equitable access to MT that is culturally sensitive is critical for optimizing therapeutic outcomes for all patients (142, 143).

### 5.2 Neuroscientific considerations

The interdisciplinary approach that integrates MT with neuroscience provides an in-depth understanding of the neurobiological mechanisms underlying music’s influence on brain function and behavior. Neuroimaging studies consistently demonstrate that MT engages various brain regions responsible for emotions, cognition, and motor functions. Research has highlighted that music can stimulate neuronal plasticity, enabling the brain to form new neural connections, a relevant aspect for rehabilitation and cognitive development (63, 64, 132, 144).

Furthermore, MT facilitates the release of neurochemicals such as dopamine, associated with pleasure and reward, and endorphins, which contribute to pain reduction (49, 132). An additional relevant aspect is that music modulates cortisol levels, helping to reduce stress and anxiety (145).

However, while the benefits of MT are clear, it is also essential to investigate and acknowledge the potential risks.

### 5.3 Potential risks and limitations

Integrating music technology into therapeutic practices, while offering notable advantages, presents risks and limitations, particularly for vulnerable populations like children.

#### 5.3.1 Sensory overload

One major concern is the risk of sensory overload, especially in children with neurological disorders or heightened sensory sensitivities. Complex and intense musical stimuli can lead to confusion and discomfort, particularly in children with autism spectrum disorder (146, 147).

#### 5.3.2 Elicitation of unwanted memories

Music has the potential to evoke painful memories, which can be particularly distressing for children with past traumatic experiences (148, 149).

#### 5.3.3 Physical injuries

MT activities involving movement or dance may carry the risk of physical injuries. Children with motor impairments are especially vulnerable to falls or injuries if the activities are not adequately supervised and adapted to their physical capabilities (150).

#### 5.3.4 Privacy and data security

Collecting and archiving sensitive patient information requires particular attention to ensure privacy and data security. It is fundamental to ensure that data is securely stored and anonymized, adhering to regulations such as the General Data Protection Regulation (141).

#### 5.3.5 Economic disparities

Music technologies for electronic composition can be expensive, limiting access to services in economically constrained settings. Ensuring equitable access to MT resources is essential to reduce disparities (151–153).

#### 5.3.6 Photosensitivity and epilepsy

Multisensory environments, including visual stimuli, may trigger seizures in children, particularly those with epilepsy. Adapting the therapeutic environment to avoid such triggers is essential (154).

#### 5.3.7 Age-related considerations

Age-specific factors influence session duration and frequency. Younger children may require shorter, more frequent sessions, while older children may benefit from longer, structured sessions (155).

#### 5.3.8 Technological complexity and accessibility

The complexity of music technologies can limit access, especially in under-resourced settings. Over-reliance on technology may also reduce direct human interactions (153, 156).

#### 5.3.9 Psychological implications and emotional challenges in recording the maternal voice

The recording of the maternal voice, essential in the EM MT intervention, may induce significant discomfort in the parent. This discomfort often stems from the embarrassment of singing in front of others and the challenges of meeting the specific technical demands set by the music therapist. The intimate nature of the process, which requires the use of the voice in a highly structured context with high technical expectations, accentuates these challenges. This process can trigger intense emotions, with reactions such as crying, further complicating the recording. To manage the parent’s stress and emotions, coping strategies are fundamental. An empathetic and supportive approach, including emotional validation and relaxation techniques, can create a more serene therapeutic environment. Additionally, gradual preparation and the use of mindfulness can help parents focus on the present, alleviating anxiety and improving the quality of the therapeutic interaction. These measures not only adhere to ethical principles but are also essential for optimizing treatment efficacy (157).

#### 5.3.10 Challenges for the training of music therapists in the Euterpe method

The effective implementation of the EM requires highly specialized therapists trained in MT, neuroscience, and musical technologies. This necessity may limit the availability of qualified professionals, particularly in resource-limited areas. Furthermore, the ongoing advancement of musical technology and therapeutic techniques necessitates continuous education and training, creating potential barriers for new professionals entering the field. Therefore, it is essential to develop interdisciplinary training programs and continuing education pathways that ensure adequate preparation and equitable access to training for therapists.

## 6 Ethical considerations

In conducting pediatric research, it is essential to implement rigorous ethical protocols to manage power dynamics and protect the rights of participants. We ensured the active involvement of children and parents, fostering a collaborative and respectful environment. Informed consent was obtained from parents, ensuring they were fully informed about the research objectives, procedures, and potential risks.

During the MT sessions, safety measures were adopted to protect participants by monitoring and adjusting therapeutic activities based on their reactions. The confidentiality of data was assured through anonymization and restricted access to sensitive information. Additionally, the active participation of children was emphasized, respecting their autonomy and individual preferences.

The research team engaged in professional supervision and critical reflection sessions to address ethical issues and ensure that therapeutic decisions adhered to the highest standards of integrity. This approach ensured that therapeutic practices not only respected the ethical principles outlined in the Declaration of Helsinki but also contributed to improving the quality of life for patients while maintaining high standards of scientific and human responsibility.

## 7 Discussion

The EM approach presented in this study represents an innovative integration of musical sciences, MT, and pediatric care. Through the use of electronic compositions and multisensory environments, a marked improvement in therapeutic outcomes emerges, specifically addressing the needs of pediatric patients. Technology allows for real-time adaptability, making treatment flexible and dynamic while optimizing therapeutic procedures.

Our previous studies (35, 36) have demonstrated the effectiveness of the EM approach, highlighting tangible progress in emotional regulation and sleep quality in children, as well as reductions in parental stress and improvements in family quality of life.

The multisensory integration has further enriched the intervention, enabling a more comprehensive response for patients with specific needs, such as those with disabilities or sensory disorders. The personalization of musical compositions has enhanced patient engagement, promoting autonomy and self-esteem.

Active involvement of parents has made the intervention even more effective. The adoption of techniques in daily family routines has strengthened the parent–child bond and improved treatment efficacy without requiring specialized training for caregivers.

The analyses by Paterson et al. and Le Vu et al. (39, 41), which examined our studies (35), emphasized the importance of the replicability of the EM protocol and its capacity to respond to emerging medical needs, such as telerehabilitation. This article rigorously addresses these aspects, proposing algorithms to ensure effective methodological application, even remotely.

Moreover, the use of multisensory therapeutic environments has shown strong potential in improving patient communication and participation, facilitating pediatric rehabilitation within an integrated sensory stimulation context.

This study demonstrates how the integration of medicine, neuroscience, and MT through the EM approach can significantly enhance the well-being of pediatric patients and their families. Furthermore, the results suggest new directions for developing increasingly personalized and interdisciplinary clinical interventions.

## 8 Implications for practice

### 8.1 Clinical applications

The findings of this study reveal significant implications for a wide range of clinical and institutional settings. EM represents a highly detailed non-pharmacological intervention that integrates innovative technologies and personalized compositions, both pre-programmed and real-time, to address the specific therapeutic needs of patients. This innovative approach is particularly suited to enhancing patient care across diverse settings, promoting emotional and psychophysiological health and well-being.

### 8.2 Key application contexts

EM can be applied in a variety of settings, including pediatric hospitals, rehabilitation centers, psychiatric institutions, facilities for degenerative diseases, post-stroke rehabilitation units, oncology and palliative care centers.

### 8.3 Neurobiological mechanisms

The integration of multisensory stimulation, a key element of EM, interacts with the central nervous system, promoting neurobiological regulation that supports neuroplasticity and functional recovery. Recent studies have shown that multisensory stimulation activates distributed neuronal networks, facilitating sensory information integration and enhancing modulation of the patient’s emotional and cognitive states. Specifically, the combination of visual, auditory, and tactile stimuli can enhance synaptic plasticity in higher cortical areas, contributing to improvements in cognitive and motor skills (63, 64, 71, 132, 144, 158).

EM may also positively influence neuropsychological circuits involved in anxiety regulation and stress management, increasing therapeutic efficacy while reducing the need for pharmacological interventions (132, 145).

These mechanisms underscore EM’s importance not only as a tool for physical rehabilitation but also as an intervention for managing emotions and promoting psychological well-being.

### 8.4 Case study 1: Pediatric hospitals and rehabilitation centers

In a pediatric hospital, a seven-year-old child with cerebral palsy manifested emotional dysregulation toward his mother, evidenced by marked emotional indifference and intolerance to the mother’s voice. A significant change was observed after 3 weeks of EM treatment. The mother–child relationship showed remarkable improvement, with increased affective interaction and a richer emotional response from the child. This suggests that EM is effective in promoting psychological well-being and improving relational dynamics, facilitating affective interactions and emotional communication between child and mother (36).

### 8.5 Case study 2: Oncology centers and palliative care

In an oncology center, a six-year-old girl with leukemia benefited from EM MT in reducing anxiety and managing pain during chemotherapy. The combination of MT with a multisensory environment significantly improved her mood and general well-being, highlighting EM’s potential in integrated therapeutic support (159).

### 8.6 Future research and perspectives

To further optimize EM, focused research on sensory integration mechanisms is essential, exploring the impact of orchestral therapy on cognitive function, motor skills, and social inclusion. Orchestral therapy, in particular, offers opportunities to improve discipline, responsibility, and teamwork, with positive effects on neuroplasticity and social integration, reducing isolation and promoting emotional well-being (160).

EM is confirmed as an innovative and resilient therapeutic approach that extends MT’s applicability to complex clinical settings. Its ability to synergistically integrate with other therapeutic modalities and its customizable adaptability throughout the treatment pathway constitute a substantial advancement in improving patients’ quality of life.

## 9 Conclusion

The integration of neuroscience, music, and technology has led to the development of personalized therapeutic interventions, emphasizing the importance of an innovative approach in pediatric rehabilitation.

The EM approach has proven effective in improving critical aspects of children’s health, including sleep quality, emotional regulation, and reducing parental stress, contributing to the overall well-being of the families involved.

The combination of personalized electronic compositions with multisensory environments has enhanced the effectiveness of treatment, leading to more significant outcomes. Moreover, the active participation of parents has been an essential factor in the rehabilitation process, improving engagement and the efficacy of therapies.

The results obtained provide important practical implications for the replicability of the EM approach in clinical settings, suggesting that its application can enrich pediatric rehabilitation programs. The findings of this study not only expand the understanding of MT in treating developmental disorders but also lay the groundwork for further research in the field, aiming to explore its efficacy and establish clinical practice guidelines.

## Data Availability

The original contributions presented in the study are included in the article/Supplementary material, further inquiries can be directed to the corresponding author.
