# Supplementary material for: Euterpe music therapy methodology and procedure algorithms
Source: Front Neurol. 2024 Oct 28;15:1443329. doi: 10.3389/fneur.2024.1443329 (PMC11551122; doi:10.3389/fneur.2024.1443329)
Supplement: Supplementary file 1 [file Presentation_1.pdf]

Supplementary Material

The Supplementary Material aims to provide a detailed overview of the technical aspects of the setups, an in-depth description of the software used, and comprehensive guidelines for creating personalized therapeutic compositions. The objective is to facilitate the replicability of the protocol and ensure that other professionals can effectively adopt and adapt these techniques in their therapeutic practices.

S1. Setting

During the pre-treatment phase, the music therapy team sets up the environment. This involves executing interventions to manage various software and audio, video, and image devices, as well as the lighting. Specifically, the wiring and control of these devices are carried out as shown in the electrical diagrams.

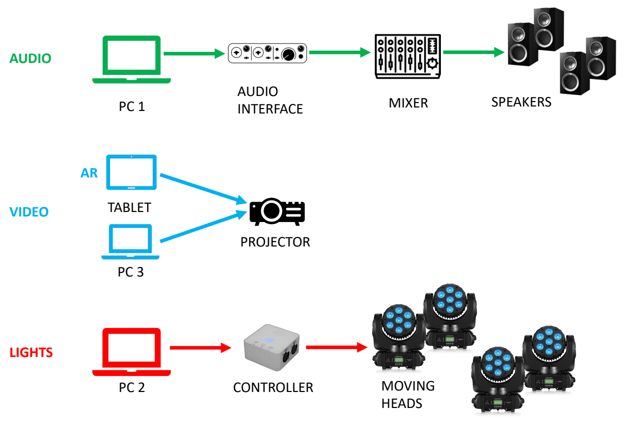

Supplementary Figure 1. Audio, video, and lighting signal management devices.
The figure shows the paths of the audio, video, and lighting signals separately, with the connections to the corresponding devices.

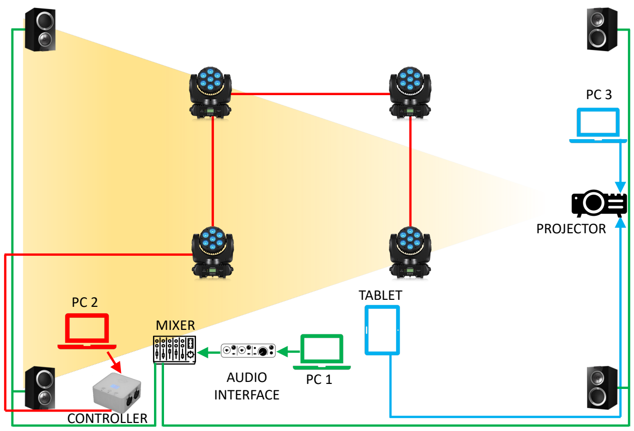

Supplementary Figure 2. Wiring of audio, video and lighting equipment in the Synesthesia Room.
The arrangement shown allows the music therapist to manage the three signals directly from the DAW workstation. It is important to note that the video signal is managed remotely through dedicated applications and devices.

S2. Materials and Equipment

2.1 Audio Hardware

The audio configuration of the Synesthesia Room is designed to modulate the patient's auditory stimuli in different spatial orientations. The signal output of the computer is connected to the audio interface. Both condenser (ceiling mounted) and dynamic microphones are integrated into the interface to capture panoramic and vocal recordings. The first four outputs of the audio interface go into the first four channels of the mixer. These mixer channels are panned and routed to two output paths: the primary Main Out and the secondary Group 1-2. The remaining are routed to electronic musical instruments such as slapstick, theremin, keyboard, and electronic drums. These signals can be processed via internal mixer effects such as reverb, delay, chorus, echo, and flanger.
The dual diffusion system facilitates sound movement between sources, allowing two-sided, right-left, and cross-shaped configurations.

2.2 Audio software management

The utilization of various audio software is necessary to develop compositional processes with diverse technical characteristics. We list and describe the features of a range of audio software that enable us to achieve performance aligned with methodological and therapeutic objectives.

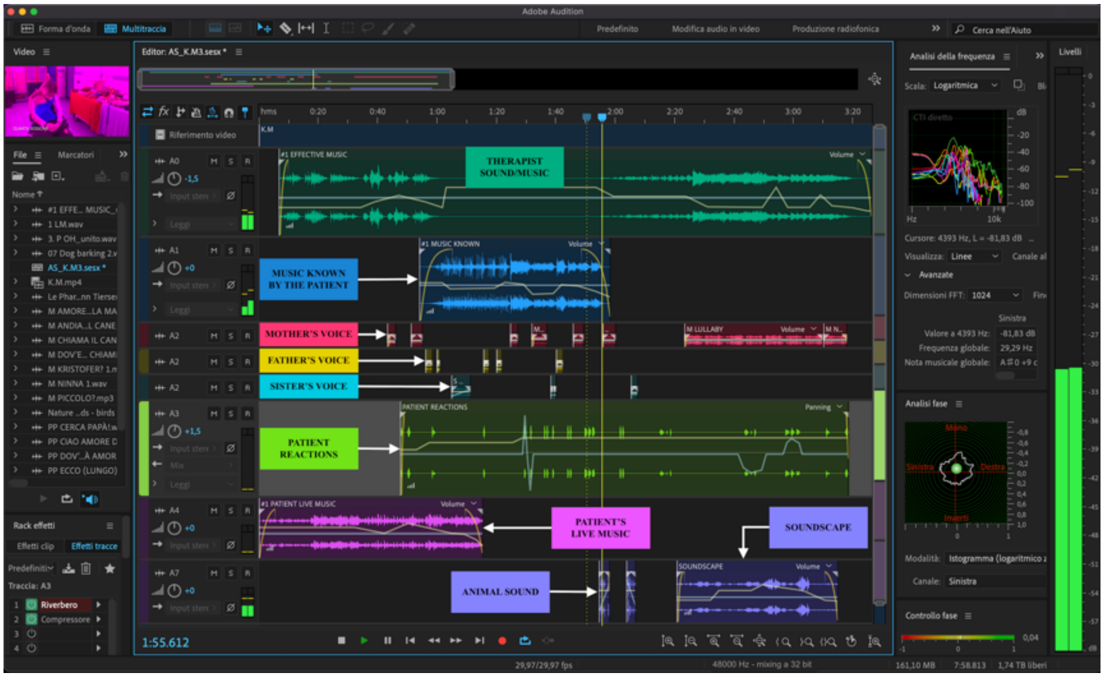

Supplementary Figure 3. Adobe Audition. The image shows an example of PTC.

Adobe Audition, a software dedicated to the recording and the editing audio developed by Adobe Inc., offers a comprehensive toolset encompassing multitrack, waveform, and spectral visualization functionalities. This facilitates the creation, mixing, editing, and restoration of audio content. Its integration with Adobe Premiere Pro optimizes workflows across both audio and video productions.

We employ Adobe Audition for generating Archive A audio clips, managing sound during sessions, and creating Personalized Therapeutic Composition (PTC).

Pro Tools, developed by Avid Technology, serves as audio editing software for recording, mixing, and audio post-production, primarily in music and video industries. Avid's suite fosters workflows encompassing audio production with Pro Tools, notation via Sibelius, and video production with Media Composer. Pro Tools is utilized for recording and acquiring musical scores. Live recordings are transposed to MIDI to extract music sheets through the Score Editor window. Music sheets are further quantized and refined with Sibelius or equivalent notation software. The software interface exhibits the Edit window along with the Score Editor window at the bottom. The waveform recording of the initial track (highlighted in red) undergoes conversion to MIDI format (depicted in orange) within the third track. The Score Editor (highlighted in blue) displays the musical notation of the third track, enabling live performance during sessions.

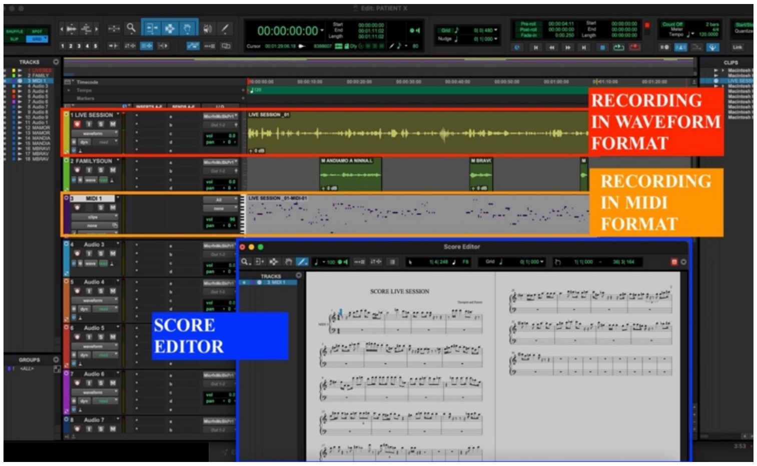

Supplementary Figure 4. Pro Tools.

FMOD Studio (free download software), developed by Firelight Technologies, provides cutting-edge tools for interactive audio, primarily leveraged within the gaming industry. Its software-mixed architecture, extensive cross-platform support, and sound design utilities make it invaluable. We use FMOD Studio to create sound narratives and to orchestrate the randomized and parameterized playback of Archive A files or other audio elements. The image presents the library of files (Assets) on the left axis, while the center displays the Event Editor, showcasing Loop Playback, the Timeline, and all tracks. Sound design is managed in different phases, transitioning from individual clip reproduction (blue clips) to regions where multiple clips play simultaneously using functions like Scatter (depicted in green in track 4) or Multi Instruments (shown in dark green in track 3). Different parameters interact with spatial perception. These allow the regulation of the location of sound sources, facilitating the creation of diverse environmental scenes. On the right axis, the 3D Preview tool enables sound panning: the white dot, movable with a cursor, simulates the movement of the sound source in space,

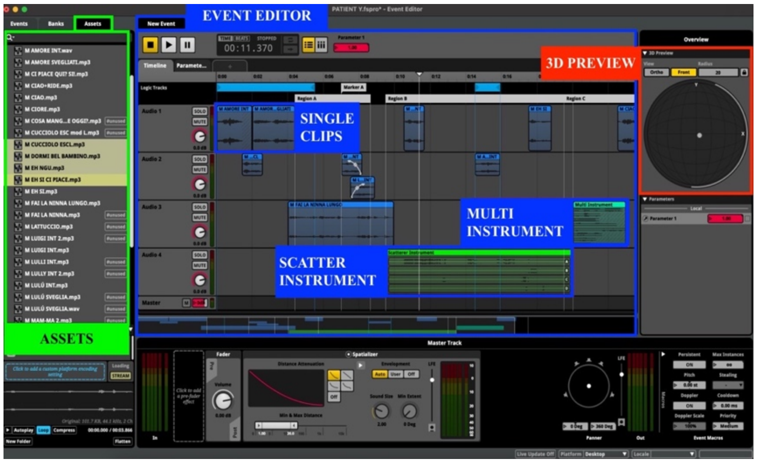

Supplementary Figure 5. FMOD Studio.

while the gray line on the circumference demonstrates how the sound is panned to the speakers simultaneously with its movement. Directly below, the Parameters tool governs the playback behavior of Sounds and Events.

MPC Beats, from Akai Electric Company Ltd. serves as a beat-making software with drum programming, sampling and audio recording functionalities. Its library of samples, loops, and built-in virtual instrument plug-ins makes it easy to create a variety of musical genres. This software is used directly by patients during sessions. Patients interact with a 4x4 grid via a touch screen, triggering sounds preset by the therapist based on the patient's preferred musical genre. The image depicts the Inspector panel on the left axis, providing audio or MIDI information regarding the sequence, track, and program of the selected "House" musical genre template; below are the channel strips for monitoring and mixing tracks. In the center lies the Grid Editor, showcasing tracks and notes (represented by colored rectangles) along the timeline. Above the keyboard, the highlighted 4x4 grid corresponds to each sound (template sample) configured in the channel list. Patients engage with these sounds via a touch screen, promoting motivation, motor skills, and oculo-manual coordination. On the right axis, the browser displays the library contents, encompassing all templates across various music genres.

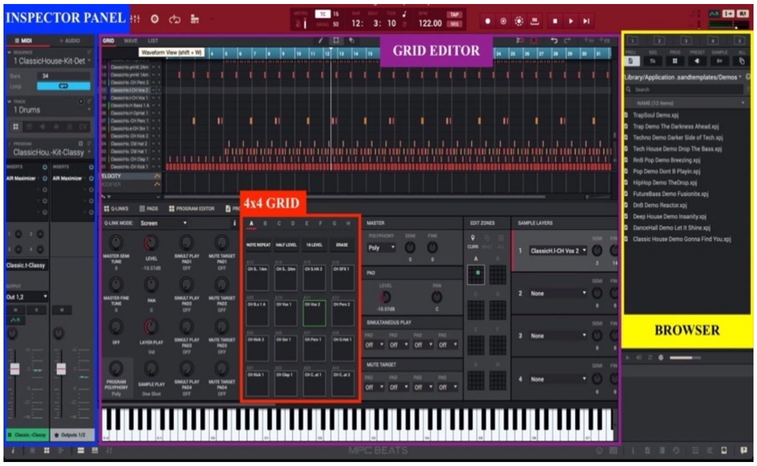

Supplementary Figure 6. MPC Beats.

Ableton Live, developed by Ableton AG, is a sequencer widely used by DJs for composing, recording, arranging, mixing, and mastering music. We use Ableton Live to create personalized sessions composed of audio clips from Archive A or the music therapist's sound bank. These sessions are designed for patient interaction, facilitated by the Akai APC Key 25 controller. The software interface displays the Browser on the left, the Live Set (project) on the right, featuring session tracks housing up to five clips each, and at the bottom, effects tailored to the patient's perception. The personalized session is directly controlled by the patient using the Akai APC Key 25

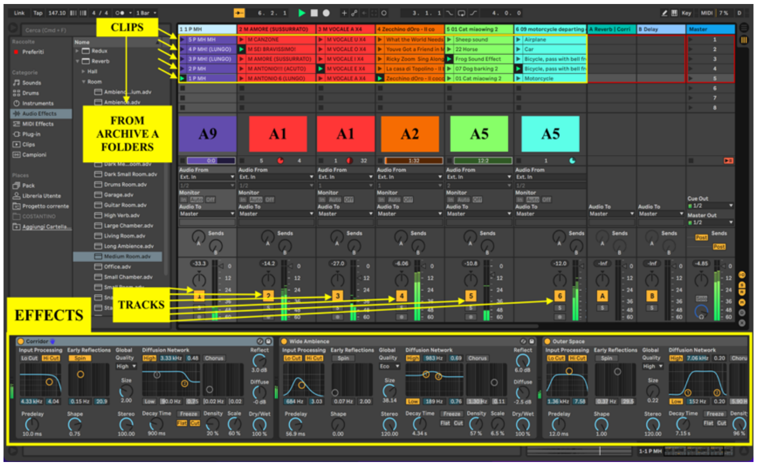

Supplementary Figure 7. Ableton Live.

controller. This controller features a 5x8 multicolor clip launch-grid enabling the patient to trigger preconfigured sounds. Moreover, it incorporates a two-octave keyboard for clip modulation. During the Live Set programming phase, audio files undergo MIDI conversion. The 25-note keyboard, coupled with Oct Up and Oct Down buttons, provides control over ten octaves spanning two-and-a-half intervals. Positioned in the upper right-hand corner, the 8 knobs facilitate manual adjustment of diverse parameters, including volume by default, as well as pan, send, and device controls.

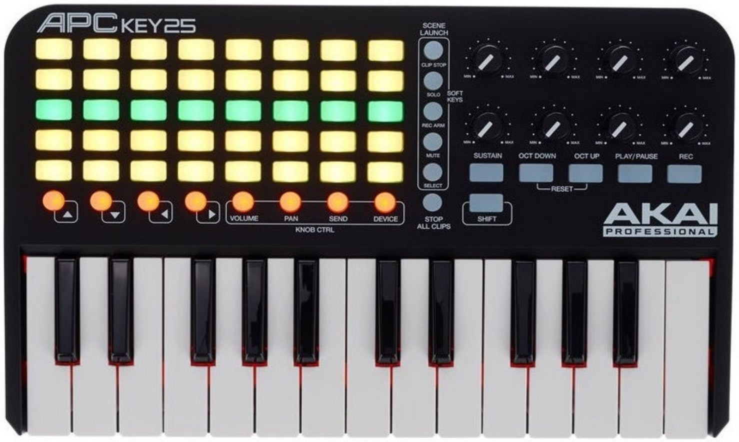

Supplementary Figure 8. Akai APC Key 25 controller.

2.3 Video Management

EM includes comprehensive session video recording for detailed analysis, monitoring of therapy progress, and capturing patient responses, images, and video clips that are essential for creating the PTC and Audiovisual Soundtrack (AVS). A variety of cameras, both fixed and mobile, are used for this purpose.

Fixed cameras, strategically placed throughout the therapy room, capture panoramic video footage during sessions. These cameras are equipped with high-fidelity sensors capable of 4K resolution recording and HDR technology for accurate color reproduction and extended dynamic range. Built-in fixed 120° XY stereo microphones provide precise audio record with optimal directionality.

Mobile cameras are used for close-up and detail shots, providing flexibility to capture multiple angles and record in 4K. Equipped with mid-side stereo microphones, they record both audio and ambient details while recording video.

While free software such as iMovie has been used initially, we recommend Adobe Premiere Pro, a leading video editing program used extensively in the film and television industry. With a comprehensive suite of tools for video editing, adding visual and audio effects, color correction, and audio sync, Adobe Premiere Pro offers a sophisticated editing experience. Seamless integration with Adobe Audition enables an efficient workflow between video and audio editing.

Projectors connected to laptops and tablets make it easy to show video content during therapy sessions. Laptops can be used to play back stored video clips and perform specific tasks, while tablets can use augmented reality to enhance the patient's experience during therapy.

2.4 Light management

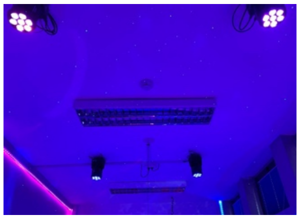

Supplementary Figure 9. Moving heads.

The music therapist manipulates the intensity, size, and color of the lighting in the Synesthesia Room through both static and dynamic (in motion) scenes, either organized or random. This approach creates new perspectives and boundaries that captivate the patient's interest, extend attention span, and enhance visuoperceptual, visuomotor, and visuospatial skills, thereby increasing motivation. The moving heads are strategically placed at four points on the ceiling, spaced 2 x 1.2 meters apart, to stimulate motor skills. These placements encourage head movements such as rotation and upright positioning, thus promoting longer periods of upward attention.

The management of these lights is organized into various scenes, which are stored presets that can be recalled via the ADJ MyDMX 3.0 software. Static scenes consist of fixed points in space with colors mixed to meet specific objectives. Dynamic scenes interact with sound, blending color and movement in space.

Supplementary Figure 10. Light management with ADJ MyDMX 3.0.

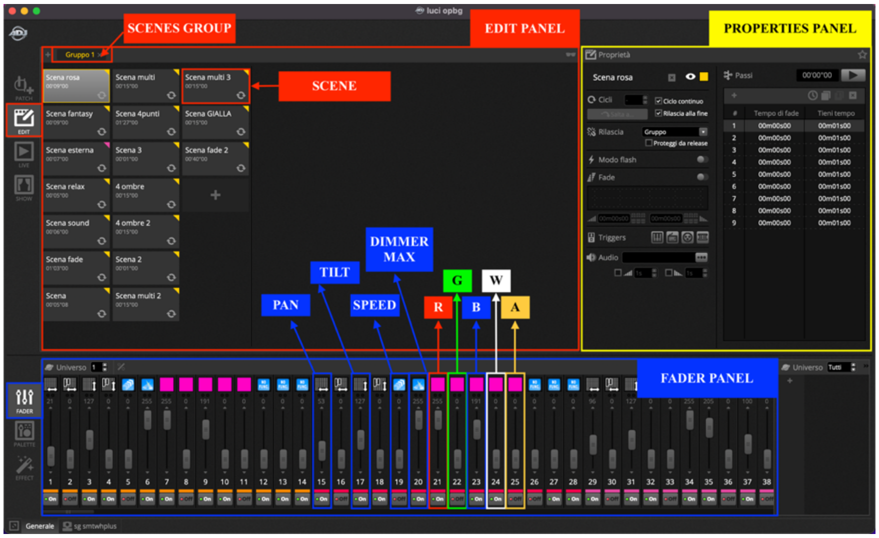

On the left side of the software interface, the Edit screen displays the grid with several scenes arranged in a group. The displayed group is organized into scenes that are designed as standardized templates that can be applied by default. Among the static scenes, there is the Pink scene, the External scene, the Relax scene, the Four-point scene or the Four-shadow scene; among the dynamic scenes, in the organized motion mode or in the random motion mode, there is the Fantasy scene, the Sound scene, the Fade scene, the Fade scene 2, and so on. On the right, the Properties panel allows you to manage and set the scene (name, number of times to play, programming of the scenes to be played automatically, fade in and fade out times, association of the scene with one or more songs). Below is the Fader panel for controlling the moving heads with the faders: Pan, Tilt, Speed, Dimmer linear, Red, Green, Blue, White, and Amber.

Supplementary Figure 11. Example of lighting.

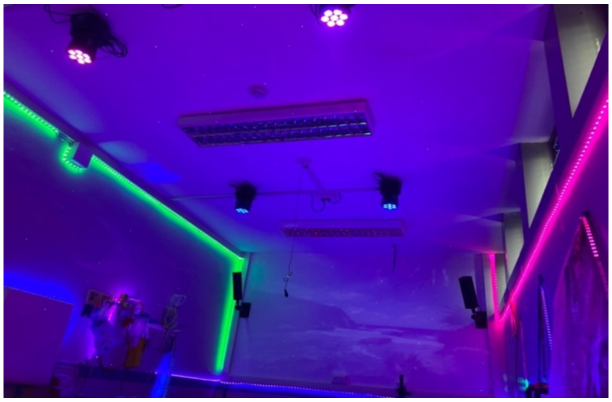

The Synesthesia Room is equipped, from bottom to top, with a variety of lighting types: two-level LED strips, projectors, fixed-light LED pars, LED pars with mobile figure projections, and moving heads. The interaction between light intensity and administered decibels is controlled via a DMX dimmer console and specialized applications. The therapist determines which sensory channel should prevail or have a reinforcing role to enhance the development of visuo-cognitive skills.

Supplementary Figure 12. Lux Light Meter Pro app.

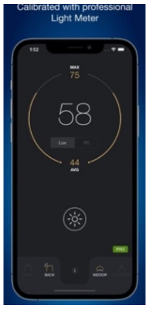

Light intensity is measured using the Lux Light Meter Pro application to gauge the patient's tolerance, which varies according to their specific pathology. Despite the important role of moving heads in the sensory stimulation provided by EM, a significant issue is the high level of background noise.

2.5 Musical instruments and tools

Both the music therapist and the patient engage in playing various instruments and tools. After assessing the sounds that are either accepted or rejected by the patient, the music therapist suggests a selection of instruments tailored to the patient's sensory and motor capabilities. This approach promotes limb motility, fine motor skills, oculo-manual coordination, head rotation in identifying the sound source, and increased tolerance to auditory stimuli.

A list of musical instruments and tools to support therapy is provided:

- Aerophones: sound balloons, recorder, transverse flute, soprano clarinet, bass clarinet, tenor saxophone;
- Chordophones: classical guitar, ukulele, violin;
- Idiophones: maracas, rattles, woodblock, shaker, xylophone, vibraphone, hang, marimbula, castanets, guiro, one- and two-tone

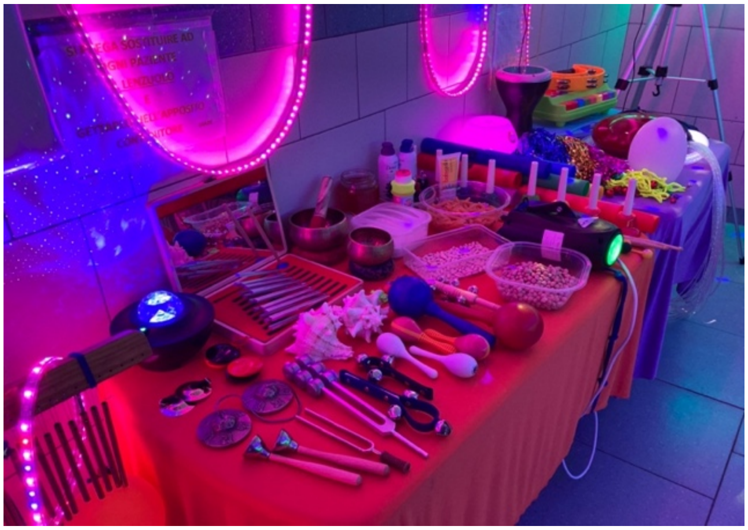

Supplementary Figure 13.
Part of the instrumentation in an example of setting.

guiro block, triangle, handbells, tubular bells, Tibetan bowls, Tibetan cymbals, tuning forks (from 32 Hz to 512 Hz);

- Membranophones: darbouka, tambourines, bass drum, gong;
- Electrophones: digital keyboard, theremin, electronic drums, slapstick, keyboard controller, MIDI grid controller.

In addition to the initial cataloging, original and classical instruments are specifically created or adapted to address the various pathologies of the patients. Examples include the multisensory net, sensory tablets, sound cloths, garbage instruments, and small membranophones equipped with rotating LED lights.

S3. Technical insights

In this section, we explore the importance of creating and populating archives in the context of music therapy. This process involves the careful collection, organization, and storage of therapeutic data, including audio, video, images, and clinical records. Techniques for iterative horizontal and vertical processes are fundamental to the continuous improvement of these archives. Horizontal processes focus on internal enhancements, while vertical processes involve the evolution of data across different levels of archives. The compositional aspects involve the creative use of archived data for therapeutic purposes, such as the creation of personalized musical compositions or the production of therapeutic visual materials. These combined approaches help to optimize the effectiveness and utility of archives in music therapy practice.

3.1 Creation of personalized Archive A, B, C, D

During the initial phase of archive creation, the music therapist carefully organizes database folders (containing audio, video, images and data) according to the personal sound history procedure to archive files from the very first session. The goal is to ensure an efficient organization that allows for quick and easy retrieval of archived clips.
The following lists and describes the contents of the subfolders within Archive A:

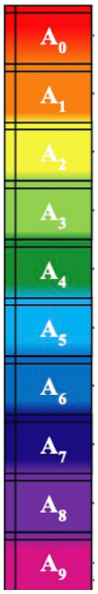

- **A_0 Therapist's Sound/Music** The music therapist creates and organizes the general musical folder. This large playlist is based on the objective characteristics of the sound (height, intensity, timbre, duration, spatiality, rhythm, tempo, agogic).
  It is designed and divided according to genres, rhythms, tones, agogics, pathologies, age, geographical origin and socio-cultural aspects. In the subdivision of playlists there are also original compositions with Adaptive Music and Sound Design techniques. These compositions are designed and created for specific therapeutic and rehabilitative purposes and are developed based on the patient's auditory sensitivity. In addition, it is important to create a personalized playlist for each patient, starting with sounds and music with characteristics similar to those reported by the mother in the anamnesis. The new compositions that are effective during the therapeutic process are added to this playlist;

- **A_1 Family Sound** This folder contains the voice recordings of the family members that the patient considers most important. In particular, there is the voice of the mother, obtained from the recording procedure during the sound history;
- **A_2 Music Known by the Patient** It stores the most important songs that the patient listens to at different times of the day, or those that have been listened to in the past;
- **A_3 Discomfort Sound** The folder contains sounds/noises that cause discomfort or stress to the patient;
- **A_4 Amniotic Sound** It stores the womb sound, heart sound, sounds/music the mother listened to during pregnancy, and environmental sounds related to where the parents lived;
- **A_5 Sound Design** The sounds of nature (soundscape - landscape), animals, tools, objects, cartoons and machines (phonosphere) are included. These clips are associated with images, memories and activities performed during therapies;
- **A_6 Electronic Noise** The folder contains the frequencies of white, pink, gray, and brown noise that cause pleasure or discomfort;
- **A_7 Body Sound** The sounds produced by the body (percussive sounds, crying, screaming, laughing) that cause amusement or discomfort are stored;
- **A_8 Live Music and Instruments Sound** The folder includes samples of instrumental sounds, timbre preferences that the patient is drawn to, recordings of live instrumental interactions between patient and therapist, and compositions created during therapy;
- **A_9 Patient Reaction** The recordings of the patient's voice and the sound responses produced during the sessions are stored.

The subfolders of Archive B are:

- **B_0 Video Archive per Session** It contains the integral videos of all the sessions, recorded by four different cameras that are positioned with different shots (three panoramas and a close-up of the patient);
- **B_1 Effective Video-Reactions** It contains video clips extracted from the integrals that are particularly relevant for therapeutic purposes;
- **B_2 Receptive Procedure Video-Reactions** It contains the video reactions related to the hospital receptive procedure (or home-based procedure) that the patient's parents send to the music therapist via an electronic device.

The subfolders of Archive C are:

- **C_0 Pictures Archive per Session** Photographs taken throughout the course of treatment are saved;
- **C_1 Effective Pictures** The folder contains the shots from which significant reactions emerge. These can be used in the realization of the AVS for the home-based procedure [33].

Therapeutic Activities Card per Session In the *Therapeutic Activities Archive D* all the sheets containing the data of the activities performed in each session are stored.

Euterpe Method S.i.M.S.

Therapist ______________________ Date ______________________

Co-therapist ______________________ Name ______________________

Session n° ____ duration ____________ Diagnosis ______________________

THERAPEUTIC INTERVENTION	THERAPEUTIC AREA	INSTRUMENTS AND MATERIALS	MUSIC BIBLIOGRAPHY	DURATION	dB	REACTIONS

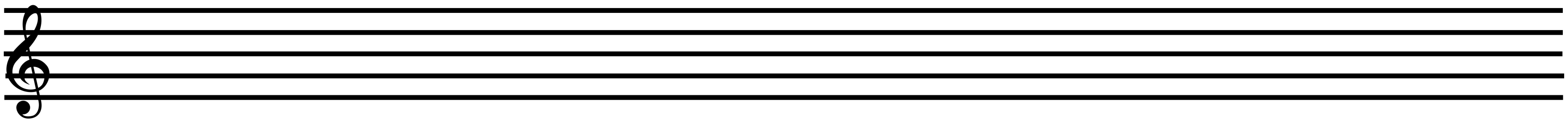

TONALITY - RHYTHM - MELODY - INTENSITY - PITCH - TIMBRE

Supplementary Figure 14. EM Therapeutic Activities Sheet.

3.2 Procedure for Extrapolating Full Raw Audio from a Full Raw Video File

In the second phase, the music therapist uses Adobe Audition software to extract audio clips from the raw recording of the mother's voice and place them in the designated folder. This procedure, aimed at implementing the sound bank for PTC processing, is carried out systematically both during therapy and at the end of each session.
The video files recorded by the cameras are exported and properly stored in the B_0 folder of the Video Archive B. The complete raw video is then imported into Adobe Audition. The Copy to New function is used to create a new copy of the entire raw audio track to preserve the integrity of the original file.

Supplementary Figure 15: Audio Extraction Process from Integral Video.

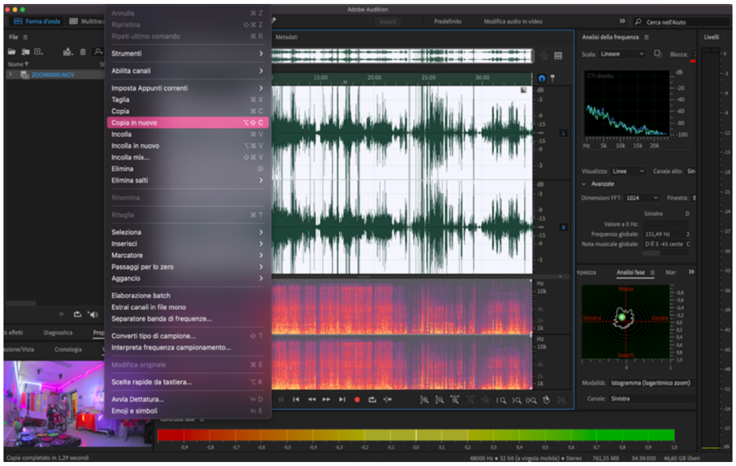

The Files panel on the left displays an imported and selected video file from a session, with a preview of the video shown below. In the center panel, the fully selected waveform is displayed, ready to be copied into a new audio file. From this waveform, vocal clips of the mother or the patient's reactions will be meticulously extracted and processed for subsequent inclusion in the subfolders of Archive A.
The therapist also extracts photographs and short video clips, which are stored in folders B_1 and C_1, respectively. These visual elements are essential for the creation of the AVS. Finally, these clips and images are uploaded to a mini-projector and used for therapeutic purposes to improve visuospatial skills, fixation times, reaction times, sight-sound coupling and lip synchronization.

3.3 Selection and Extraction of Clips from Raw Integral Audio

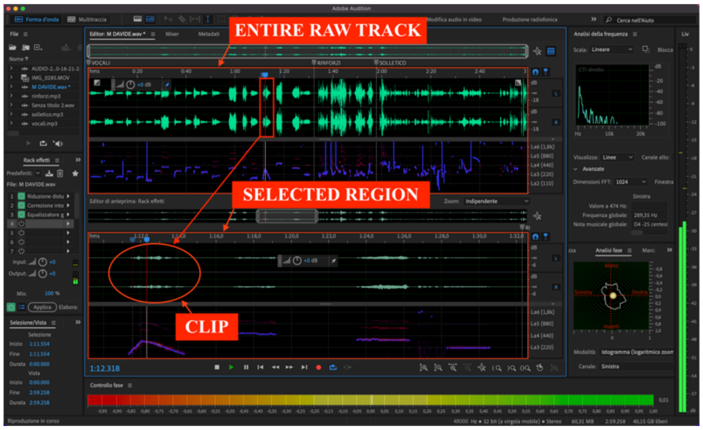

Supplementary Figure 16. A raw integral audio clip being cut.

After extracting the raw integral audio file of the session, the music therapist proceeds to the audio editing phase using Adobe Audition to obtain the vocal data underlying the therapeutic-compositional pathway. In the initial phase, time intervals corresponding to the maternal voice or patient reactions are directly selected from the waveform, fragment by fragment. The image shows a raw audio track being cut. The music therapist is extracting the audio clips from the global track that will be processed and then saved in Archive A. Clips are extracted from the entire audio track using the Copy to New function. The preview editor's display-mode interface allows simultaneous monitoring of the track's total (above) and region (below).

In the second phase, the extracted clips are initially processed with the following effects:

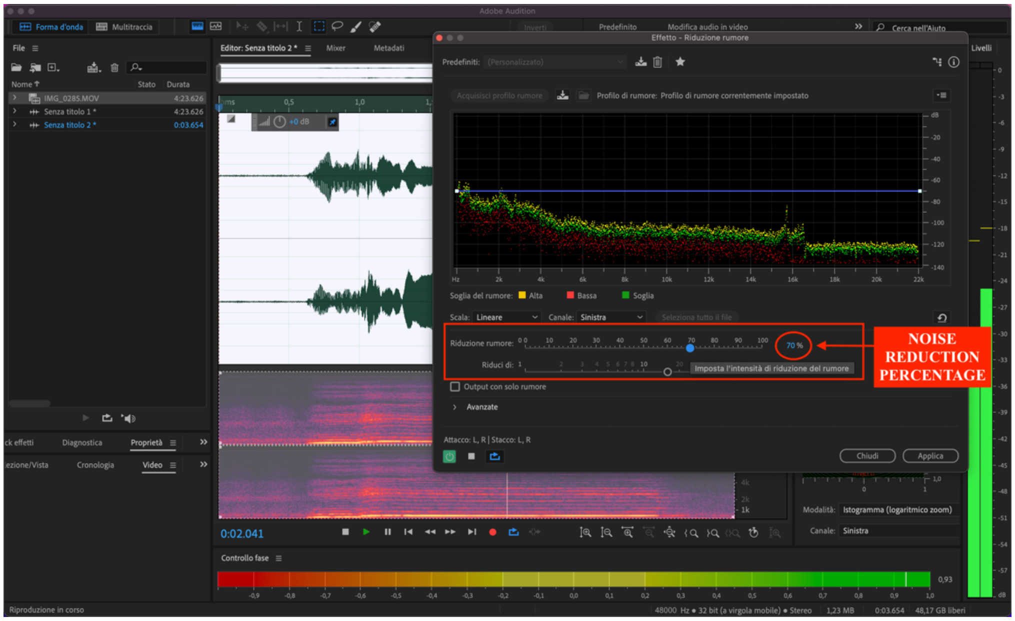

Supplementary Figure 17. First sound processing: Noise Reduction process.

Noise Reduction to reduce background noise. The image depicts a clip undergoing Noise Reduction processing. Ambient noise reduction is employed to enhance the clarity of the voice. Following the capture of the noise profile, the software generates a model utilized for removal. In this instance, the parameter is configured to 70% to minimize impact on the signal quality of the clip. The music therapist has the capability to eliminate specific frequency ranges. Control points are positioned on the control curve (blue line) based on spectral analysis of the clip, targeting the identified frequencies.

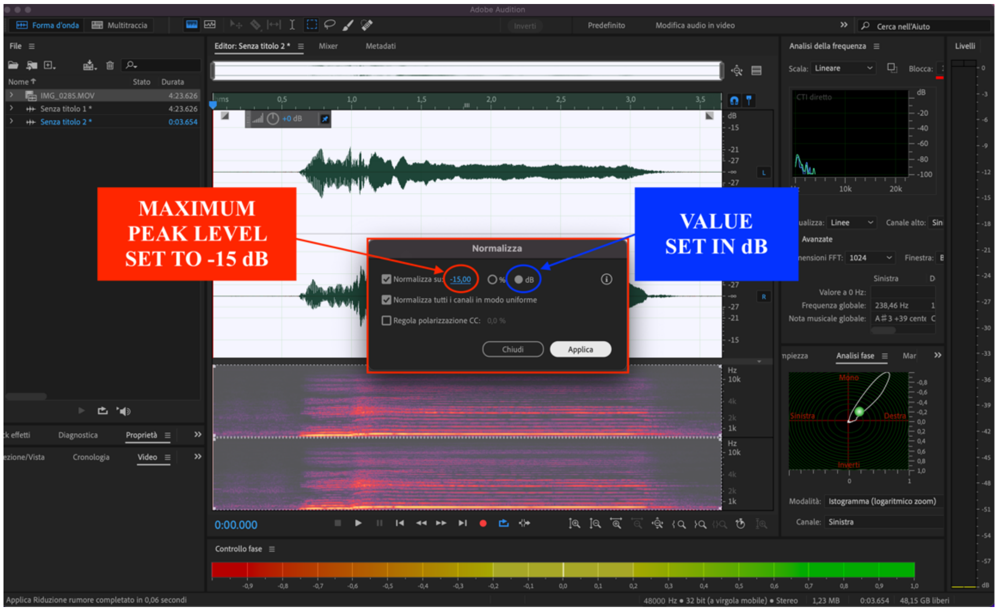

Supplementary Figure 18. First sound processing: Normalize.

Normalization to achieve balanced intensity. The effect operates on the peak level of the file, whereby the selection of the decibel value establishes the maximum intensity at -15 dB.

Any residual silence before the attack and after the release of the wave is trimmed. Then, the sound is softened with Fade In and Fade Out, and the volume is balanced by bringing the maximum amplitude level to -15 dB (dBFS). Additionally, the Graphic Equalizer (EQ) effect is used to balance the timbre across various parts of the frequency spectrum, and the Vocal Enhancer is applied to provide greater definition and depth to the voice while reducing any hissing, reverberation, and explosive sounds.
Subsequently, the speech or singing clips are scanned to obtain the different frequencies (note pitches) of the vocal range for use in live music.
The procedure is executed through frequency analysis and spectral visualization of pitch.

On the left, the image displays the waveform of the audio clip in the upper section and the corresponding pitch spectrogram in the lower section. On the right side of the software interface is the Frequency Analysis panel. Upon selecting an interval on the waveform and initiating the scanning process, the graph illustrates frequency on the horizontal axis and amplitude on the vertical axis (essential for noise reduction procedures for accurate placement of control points). The Advanced section reveals the Overall Frequency (blue box) and Overall Musical Note (green box). Additionally, the Phase Analysis panel is depicted below.

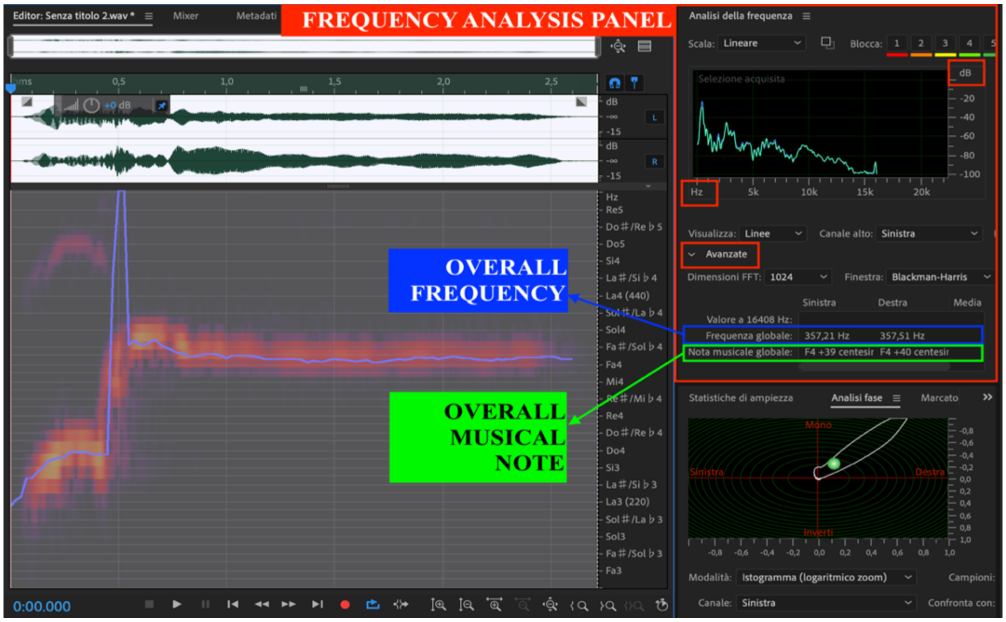

Supplementary Figure 19. First sound processing: Frequency Analysis.

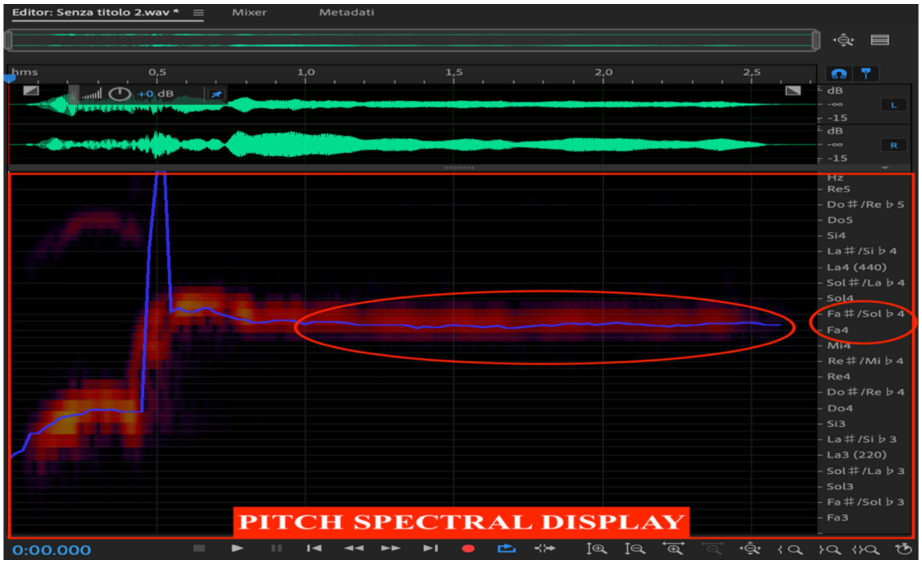

Supplementary Figure 20. First sound processing: Pitch spectral display.

In the spectral display, the fundamental pitch of the sound clip is depicted by the blue line. The vertical axis on the right indicates the pitch height (frequency) expressed in musical notes.

Before saving, clips are named based on simplicity and speed criteria to ensure recognizable sound types during the composition phase.
The synthetic cataloging must indicate the abbreviation of:

- *"Who speaks?"* e.g., mother (M), patient (P), father (F), brother (B), sister (S), grandmother (Gm), etc.
- *"What is expressed,"* describing the content (meaning of the sound) considering prosody, rhythmic elements, tone, and expression.
- Patient reaction clips are only chronologically numbered.

We report some examples: “M lullaby.mp3”, “1 P ciao!.mp3”, “F Jonh?.mp3”, “Gm I love you.mp3”, “S rainbow song.mp3”, etc.
Finally, clips are saved in stereo mp3 format with a sample rate of 48,000 Hz, 32-bit depth, and a buffer size of 1024. At the end of this process, all mother's voice files are placed in the A_1 Family Sound folder, and all patient's voice files in the A_9 Patient Reaction folder.

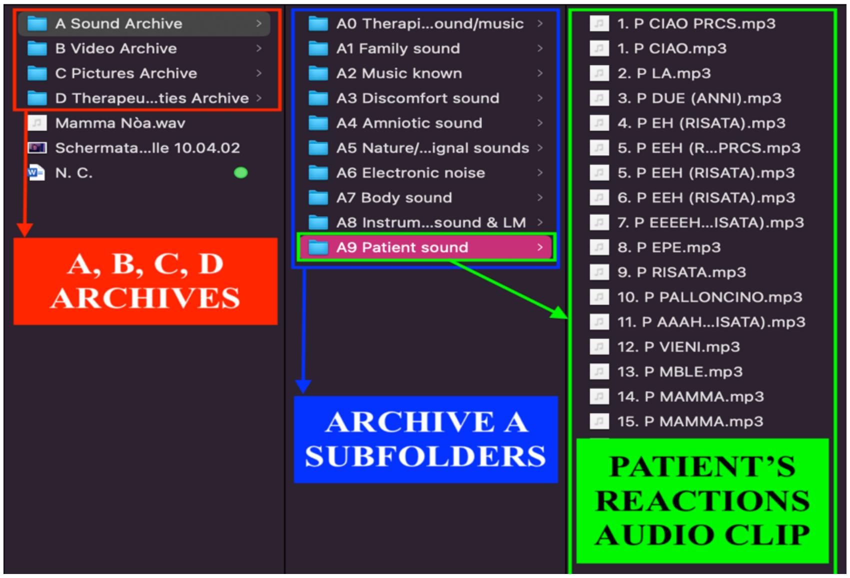

The image exemplifies storage organization. The red column lists the A, B, C, and D Archives contained within the patient's main folder. The blue column displays all ten subfolders of Archive A. The green column showcases the A_9 folder pertaining to the patient's reactions.

Supplementary Figure 21. Archiving audio clips.

3.4 Sounds processing

Clips and tracks from Archive A are processed based on patient reactions using audio editing software. Sound processing intervenes on the following parameters:

- **Intensity** Modification: Allows interaction with the proximity and distance of the sound source through volume management. The use of a sound level meter enables more precise adjustment of sound pressure based on the child's pathology (cochlear implant, hearing loss, etc.). Intensity variation can be achieved through clip Gain (digital procedure) or manual interaction with individual channel faders or the mixer master (analog procedure).
- Sound **Frequency**: Influences pitch, towards higher or lower tones, affecting the cognitive content and prosody of the clips. The music therapist applies and sets effects such as Pitch Correction, Generate Noise, Generate Tones, or Generate Speech.

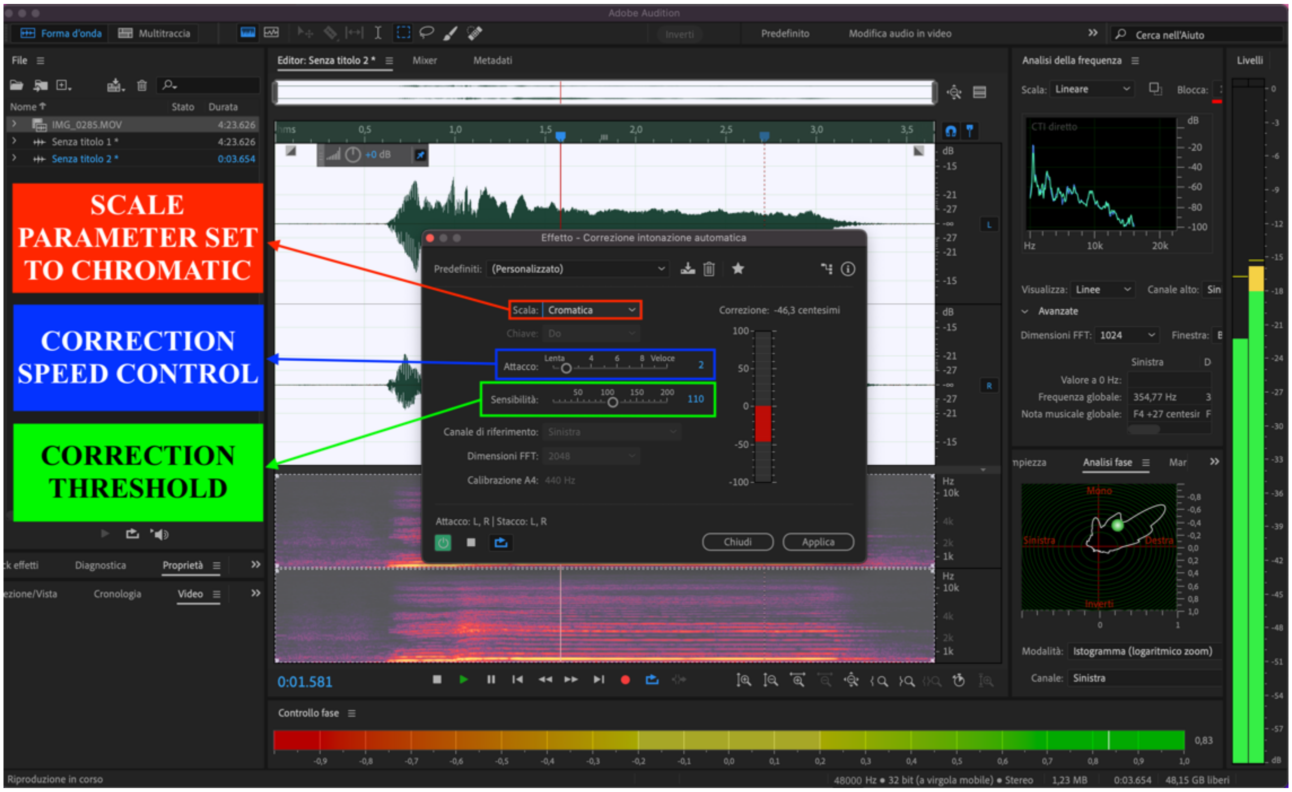

Supplementary Figure 23. First sound processing: Pitch Correction.

- The Automatic Pitch Correction effect is selected for the clip's initial pitch. The Scale parameter sets the scale type for more accurate processing, correlated with the clip's relative key. The image shows the processing of a clip using the Pitch Correction effect, in this case, chosen in Automatic mode. The Scale parameter allows the choice of the most suitable scale type for the sample between Major, Minor, or Chromatic. In this case, assigning the Chromatic mode disables the Key parameter and allows the correction of the note to the nearest one. The Attack parameter controls the quickness of correction relative to the tone of the scale. The quantity is assigned according to the duration of the notes, usually for short notes is set faster setting, and for sustained notes slower setting. Sensitivity defines the threshold, expressed in cents, beyond which notes are not corrected.

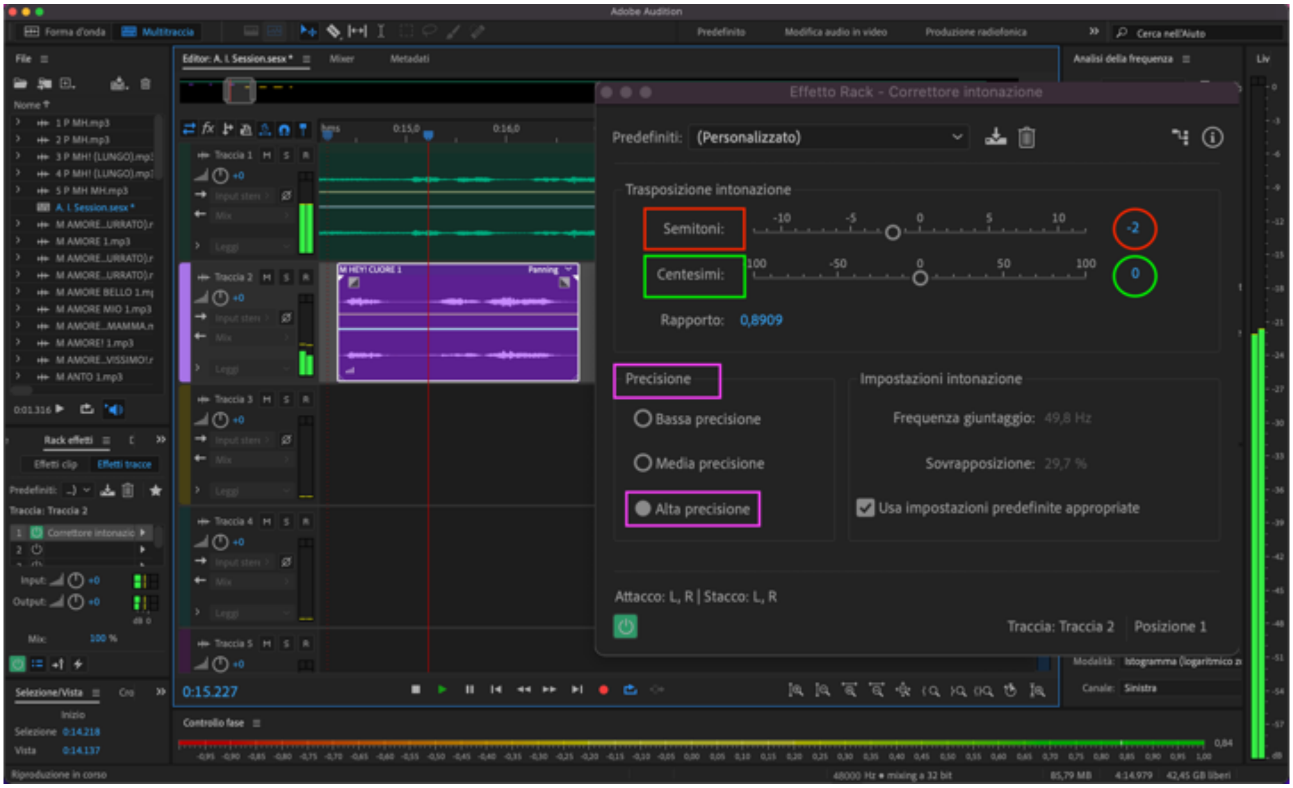

Supplementary Figure 24. Pitch Shifter effect.

○ The Pitch Shifter effect modifies the pitch (frequency) of the clip upwards or downwards using the Semi-Tones parameter. On the left, the image shows the Files panel that contains the audio clips. Below, the Effects Rack panel indicates the type and number of effects applied. At the bottom, the Selection/View panel indicates the beginning and end of the selection. In the center of the screen is the Multitrack Editor where the first two tracks are shown. The clip contained and selected in the second track (purple track) is being processed with the Pitch Shifter effect as shown by the left panel. The modification of the tonality is carried out through the Semi-Tones (red box) and Cents (green box) parameters according to the tonal system of the song in the first track. The Precision option (pink box), determining the sound quality, is set to High.

○ The Generate Noise effect transforms sound into noise. The Color parameter assigns the type of noise color (white, pink, brown, gray). The image shows the Files, Effects Rack, and Selection/View panels on the left. The center panel shows the Preview Editor with the dual-display of the clip: at the top, the entire waveform is shown, and below the resulting one from the processing with the Generate Noise. The color of the assigned noise, i.e., brown, generates a sound in which the low frequencies are emphasized.

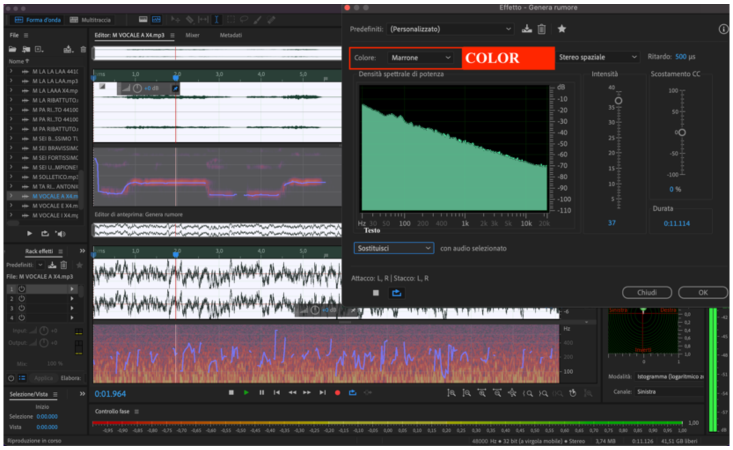

Supplementary Figure 25. Generate Noise effect.

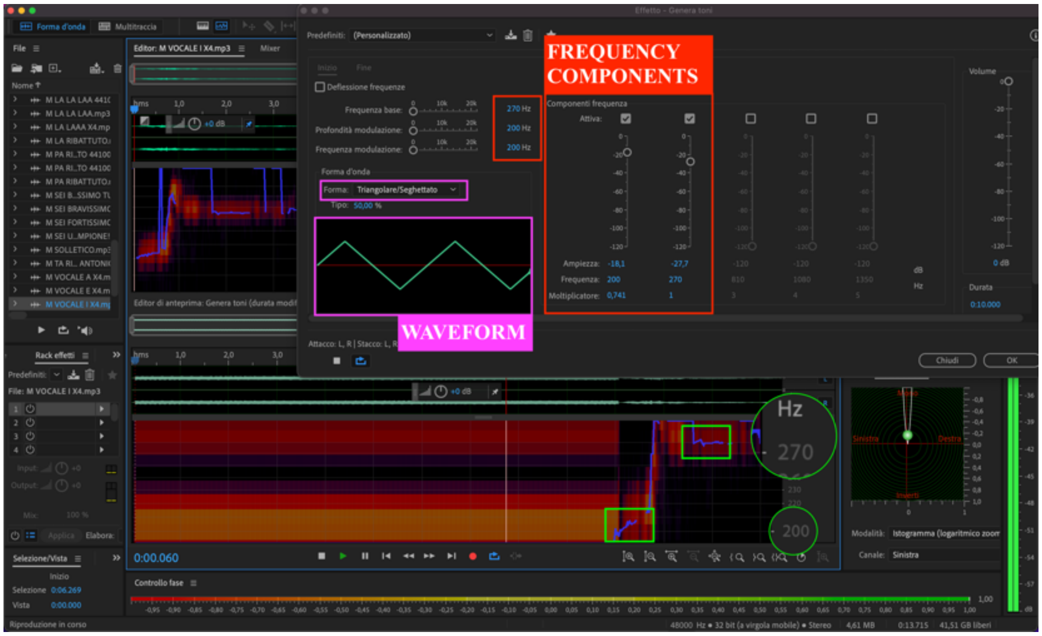

Supplementary Figure 26. Generate Tone effect.

○ The Generate Tones effect is used to convert cognitive messages into a simple waveform (musical note or chord). The screen shows the creation of a simple waveform based on the selected clip. Frequency indications of the clip, i.e., 200 Hz and 270 Hz highlighted in green in the panel below, correspond to G_3 (196 Hz) and C_4 (261 Hz) above the correct pitches. These two frequencies are the basis of this processing through the Generate Tone effect, in the upper left panel. The tone is generated by setting the two frequency indications to the parameters in the red boxes: Base frequency, where the main frequency is indicated; Modulation depth, which modulates the intonation of the base frequency over a defined interval; Modulation rate, which specifies how many times per second the frequency is modulated, producing a vibrato effect; Frequency components, which adds up to five-tone extensions to the fundamental base frequency. In the pink boxes, the type of Waveform to be reproduced is selected, in this case, a triangle/sawtooth wave produces a true triangle waveform with only odd harmonics at a Type setting of 50%.

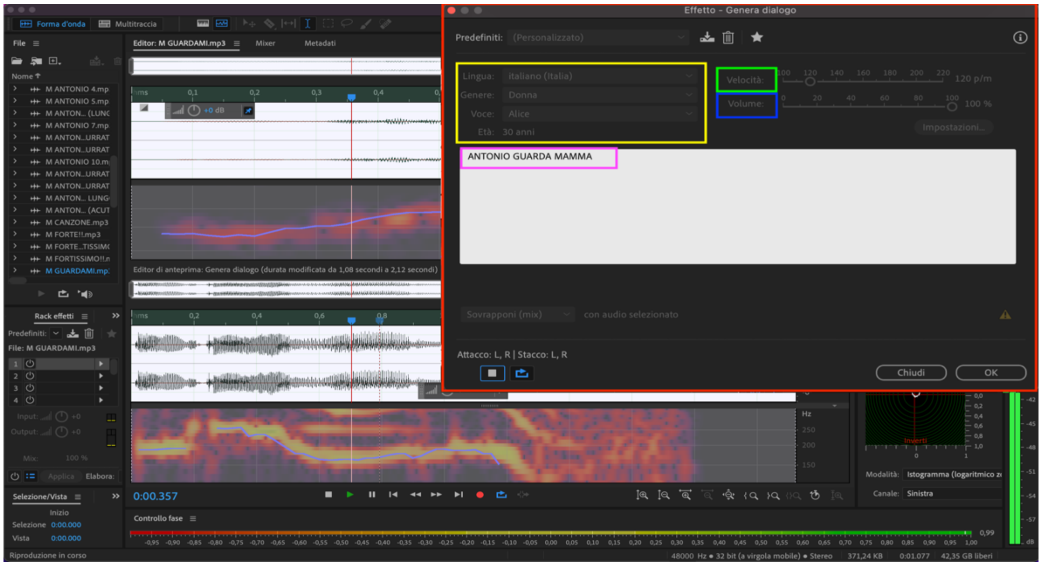

Supplementary Figure 27. Generate Speech effect.

- The Generate Speech effect creates speech synthesis files, eliminating prosody from the heard word, rendering the information purely cognitive. The Preview Editor panel in the center shows the selected clip at the top, and, at the bottom, the dialog generated by text-to-speech. The left panel (red box) shows the setting of the parameters: the choice of language, gender, voice, and age in the yellow box; the speed in the green box; the volume in the blue box; the contents of the text-to-speech file in the pink box.

- **Spatial** Perception and Sound Source Localization: Modeled through spatial effects. The therapist chooses between Reverb, Delay, Echo, or Chorus effects and decides whether to combine them. This action modifies sound color, i.e., timbre, warming it with low and mid-low frequencies or cooling it by emphasizing mid-high frequencies.

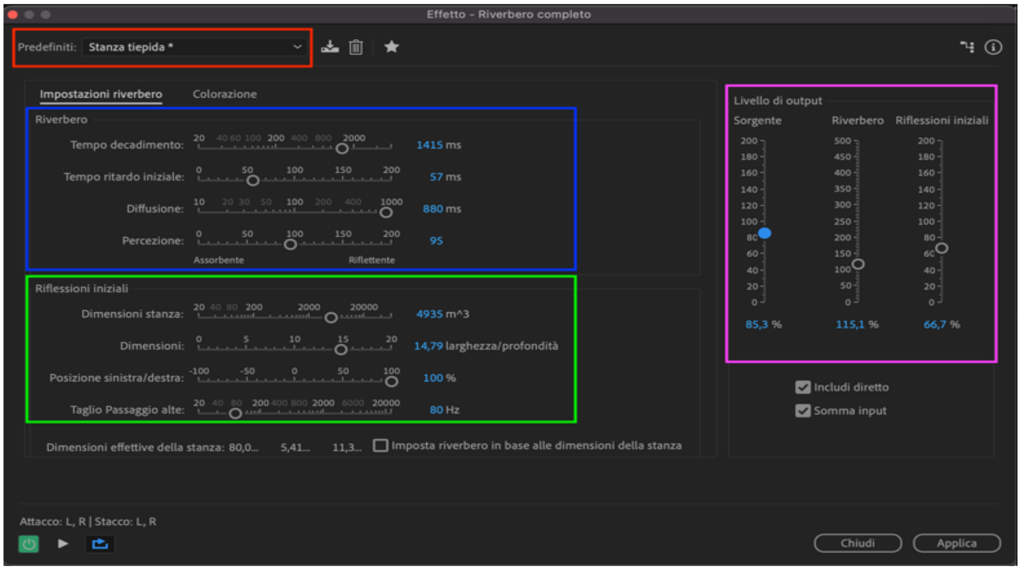

Supplementary figure 28. Full Reverb effect.

- The Full Reverb effect acts on environment contextualization. The Perception, Position, and Left/Right location parameters can be adjusted by choosing from predefined presets. The figure shows an application of the Reverb effect. The sound is processed by simulating a sound atmosphere, depending on the type of listening of the patient. In this case, the sound source is placed inside a large Warm room (red box), thus interacting with the size and type of environment. The blue box shows the quantities of the Decay Time, Pre-Delay Time, Diffusion, and Perception parameters. The green box shows the set quantities related to Room Size, Dimension, Left/Right Location, and High Pass Cutoff. To increase the reverberation effect of the clip, the Reverberation parameter has been increased to 115.1% compared to dry to 85.3% in the Output Level options (pink box).

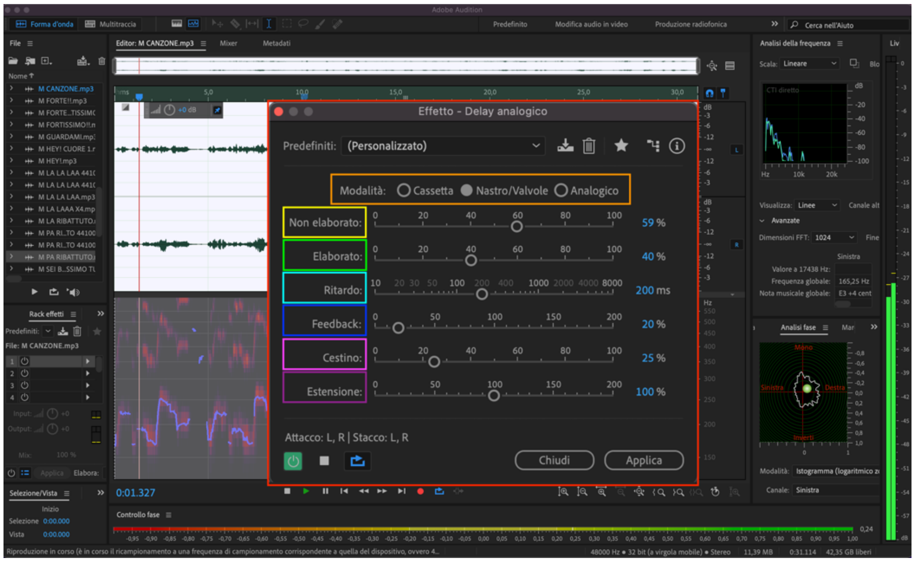

Supplementary figure 29. Analog Delay effect.

- The Delay effect allows the creation of single delays, while Analog Delay provides a warmer sound by delaying the signal without digital conversion. The amounts of unprocessed and processed audio are blended through the Dry Out, Wet Out, and Delay parameters. With the Analog Delay effect, separate copies of the original signal, repeated at intervals of 200 milliseconds (light blue box), are added to the original sound of the clip. The Mode parameter (orange box) is set to Tape/Tube which reproduces and emulates the classic effect of vintage delay units. In the yellow and green boxes, the mixed quantities of unprocessed and processed audio are indicated. The Feedback parameter (box blue) creates return echoes that slowly dissolve sending the delayed audio to 1/5 of its original volume. The Trash parameter (pink box) acts on the distortion of the signal. The increase in quantity corresponds to the enhancement in low frequencies that heat the sound consequently. The Spread parameter (purple box) allows the setting of the stereo amplitude of the delayed signal.

- The Echo effect acts on the degradation of the sound wave, influencing sound spatiality. The image shows the Echo effect panel. Parameter manipulation is set to create a ternary rhythmic figuration of the originally binary sound clip through the overlapping echoes. In addition, spatial interaction is carried out so that the sound in the environment is in motion, bouncing from one channel to another. The Delay Time parameters of the Left and Right channels have been set to 2000 milliseconds and 562.70 milliseconds; degradation is at 63.96% (Feedback parameter); the percentage of processed and unprocessed signal between the two channels in the Eco Level parameters was extremely differentiated.

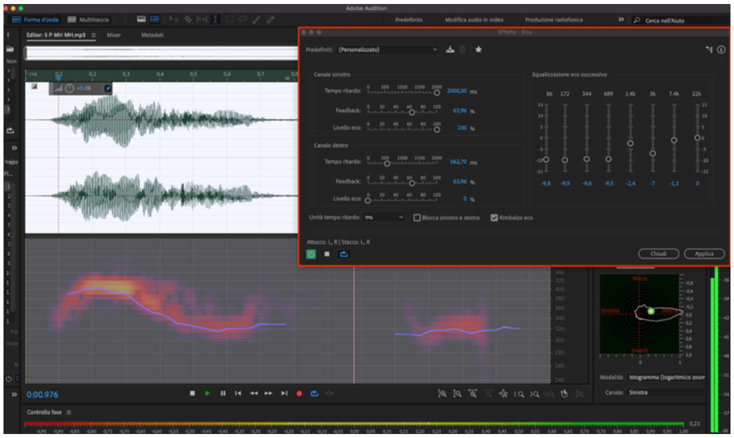

Supplementary Figure 30. Echo effect.

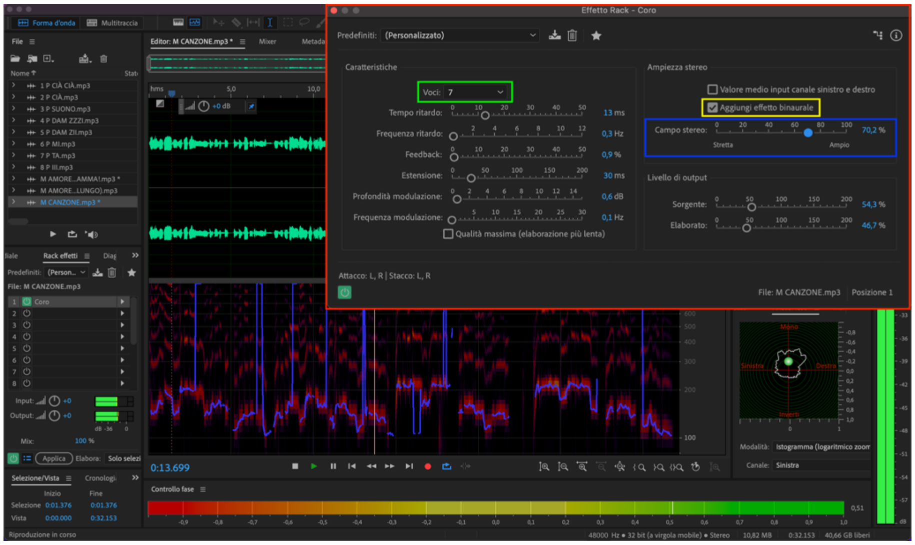

Supplementary Figure 31. Chorus effect.

- ○ The Chorus effect simulates multiple voices played simultaneously with short delays. The Voices parameter selects the number of voices to apply to the clip. The therapist also interacts with time (Delay Time), frequency (Delay Rate), and amplitude (Modulation Depth). The highlighted panel in the red box shows the application of the Chorus effect. For interaction with spatiality, more voices superimposed on the original clip are added, which have the function of enriching the sound (as indicated by the Voices parameter in the green box). The Delay Time parameter is set to create light phase shifts, and a minimum amount of Delay Rate is applied to manage their pitch over time. The placement of voices in space is assigned by the Stereo Field parameter, which is set to 70.2% so that they are moved outwards to the left and right (blue box). Clips can be further processed to add separate delays to voices in the right and left outputs by selecting the Add Binaural Cues option. This processing, however, can only be heard through the earphones, and therefore can be done for the creation of compositions to be released to the parent for receptive administration.

- Sound **Direction**: Interacts with the movement of a sound signal in space, resulting in motion. Effects that allow this are Doppler, Binaural, and Intensity Modulation.

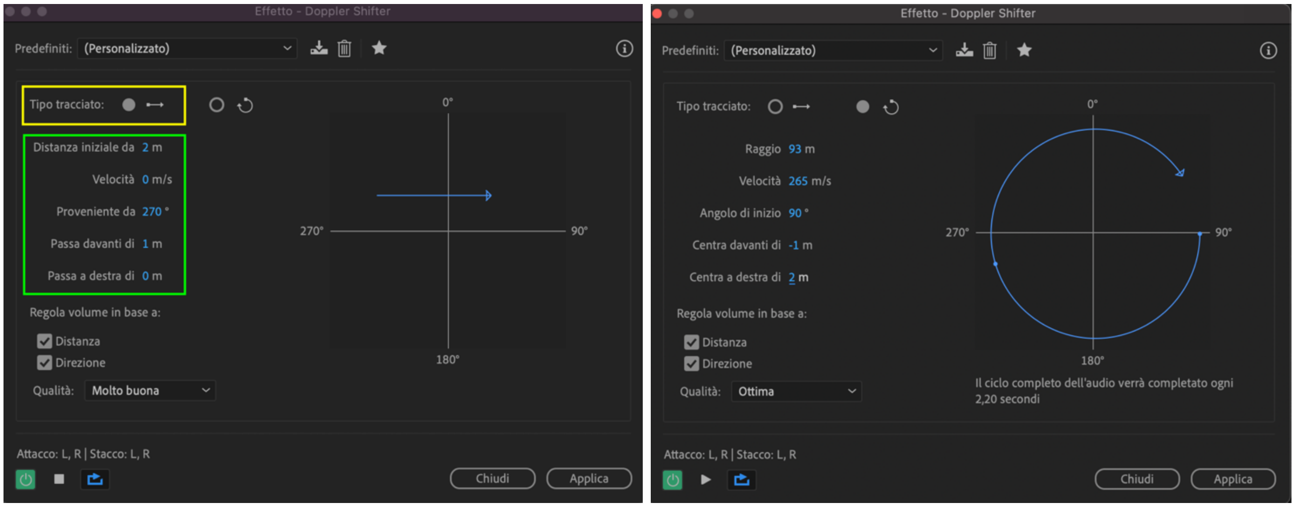

Supplementary Figure 32. Doppler Shifter effect.

- After selecting the Doppler effect and the path type, parameters that change distance (Straight Line mode: Starting Distance Away, Passes in Front of, Passes on Right of; Circular mode: Center in Front of, Center on Right of) and sound direction (Straight Line mode: Coming From; Circular mode: Starting Angle) are mixed. The panel highlighted in red shows the Doppler Shifter setting window. The type of path is selected linear (yellow box), to simulate the movement of the sound source on a linear path, from left to right and frontal to the patient. The setting of the parameters, in the green box, is made so that the progress of the source is slow in proceeding from the starting point (heard in the left channel) to the end point (heard in the right channel). On the right, the image shows an example of a circular signal path.
- The Add binaural cues box of the Chorus effect is selected to interact with the environment and the three-dimensional nature of the sound source, simulating the slight discrepancies perceived by the head and earlobes in the listening process (see Supplementary Figure 31).
- The therapist manually interacts with the mixer master faders, gradually increasing and decreasing volume, simulating the approach and withdrawal of the sound source.

- **Speed** of Sound Message: Slowed down or accelerated using the velocity parameter of the Doppler effect or with Stretch and Pitch or Clip Stretching processes.
  - The two types of paths in the Doppler Shifter process allow interaction with the Velocity parameter.
  - The Stretch and Pitch process modifies the audio signal's time through the New Duration parameter.

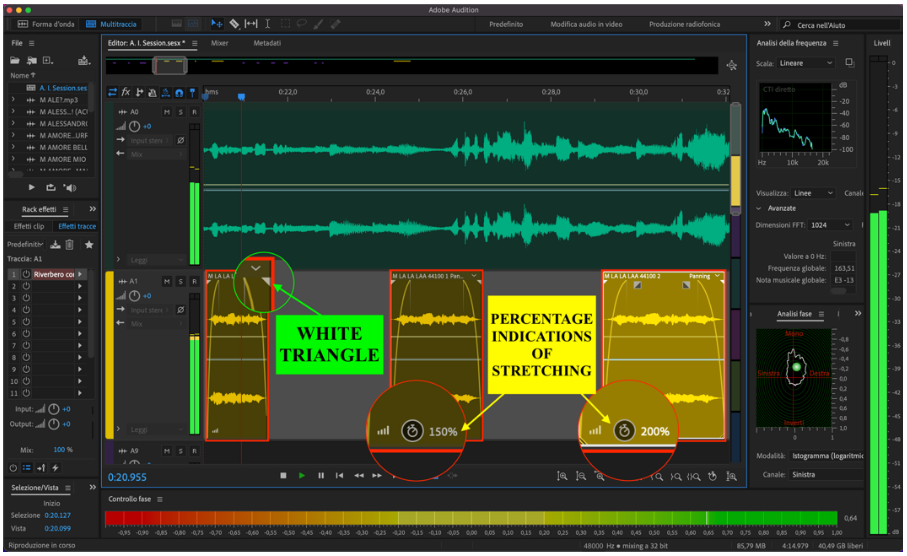

Supplementary Figure 33. Clip stretching.

Clip speed changes are possible with the Clip Stretching process only in the Multitrack Editor. At the center of the Multitrack Editor are arranged the first two tracks of the project. The first contains a song from the A_0 folder, and the second shows the audio clips from A_1 folder highlighted in red boxes. The speed parameter has been changed, dragging the triangle to the top right of the clip to the right. The red circles indicate the percentage of stretch. The three different speeds were designed according to the study of the patient's reactions.

3.5 Iterative Scheme for a Rehabilitation Process Based on a Sound Archive

An iterative process in the context of sound is a repeated processing cycle in which each phase builds upon the reactions or sound outcomes of the previous phase, progressively refining and enriching the final acoustic composition. Below, we present two legends to facilitate the understanding and practical application of the model.

Figure Type	
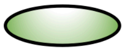	***Terminator***: Represents the entry and exit points of the algorithm, marking the beginning or end of the depicted process.
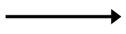	***Arrow***: Indicates the reading direction of the algorithm, guiding the logical path through the various phases or decisions of the process.
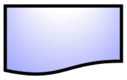	***Document***: Represents a step in the process that involves the creation of a document, generated at a different time than the flow.
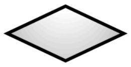	***Decision***: Indicates a point where a decision must be made. The arrows branching from this symbol present binary options, such as “true” or “false,” to represent the possible outcomes of the decision.
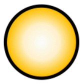	***Connector to Another Algorithm***: Allows connection to another algorithm via a connector with the same label, facilitating transitions between different sections.
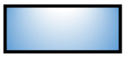	***Process***: Indicates an action, activity, or operation that must be performed.
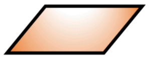	***Input/Output (data)***: Represents information or materials that enter or exit the process.
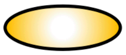	***Connector to Another Procedure***: Allows linking to another procedure external to the flow via a connector with the same label.
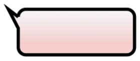	***Bubble Speech***: Indicates the question to be asked to the mother during the recording, facilitating communication within the context of interaction.
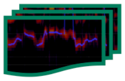	***Multiple Documents***: Represents the creation and archiving of multiple audio data generated at a different time from the main flow.
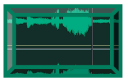	***Frame***: Represents simple or compound sound stimuli (S_n, Ss_1, Sc_n), indicating that a sound has been created or modified.

Supplementary Figure 34. The figure displays the primary symbols used in the EM algorithms, including start and end points, operations, decisions, input and output data flows, links to external procedures, and therapeutic questions, all of which are employed to visualize and guide the iterative flow of personalized music therapy.

	File Type	
	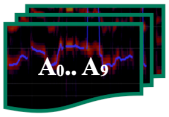	The figure refers to sound archives developed from a detailed analysis of sounds relevant to the patient. Archives $\boldsymbol{A_0}$**..** $\boldsymbol{A_9}$ are divided into 10 categories and initially contain sounds from the patient's familiar environment, aiming to personalize and optimize the music therapy pathway.
		The **blue arrow** symbolizes the input data flow, representing the stimuli administered to the patient. The **pink arrow** indicates the output data flow, i.e., the patient's responses, which are archived.
Horizontal Iterative Process	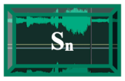	$\boldsymbol{S_n}$ denotes a numbered sound stimulus, where "n" represents the order of the stimuli (e.g., S_1, S_2). The geometric shape indicates that a sound has been created, modified, and archived.
	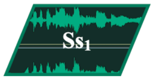	$\boldsymbol{Ss_1}$ indicates that the stimulus is simple, meaning it has not undergone any processing. It is administered as the initial input to evaluate the patient's reaction to a basic stimulus.
	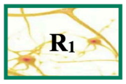	$\boldsymbol{R_1}$ is the patient's first physiological reaction to the administered sound stimulus.
	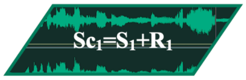	$\boldsymbol{Sc_1}$ represents a composite stimulus, generated by merging the sound stimulus (S_1) with the patient's reaction (R_1) through a recording process. This stimulus is archived and becomes a new compositional input. This horizontal process continues iteratively, progressively adapting based on the patient's responses.
Vertical Iterative Process	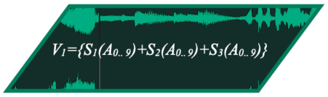	The expression $V_1=\{S_1(A_{0..\,9})+S_2(A_{0..\,9})+S_3(A_{0..\,9})\}$ indicates the fusion of three overlaid stimuli (S_1, S_2, S_3), extracted from the archives ($A_{0...9}$). This combination generates a composite stimulus (V_1), where the sound components are performed simultaneously rather than sequentially.
	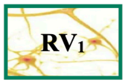	$\boldsymbol{RV_1}$ represents the patient's vertical reaction to the first vertical stimulus V_1. This reaction is a physiological or behavioral response integrated into the iterative process, influencing the subsequent generation of sound stimuli and contributing to therapy adaptation.
	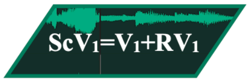	$\boldsymbol{ScV_1}$ denotes the composite vertical sound stimulus. This stimulus results from merging a vertical sound stimulus (V_1) with the patient's reaction (RV_1).
	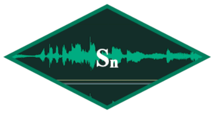	$\boldsymbol{S_n}$ in the diamond shape represents the decision point where a new sound stimulus (S_n) from the archives is introduced. If selected, the stimulus is fused with the previous composite vertical stimulus (ScV_1) to generate V_2, continuing the therapeutic process.

Supplementary Figure 35. Geometric figures and mathematical expressions describing the steps of the iterative process.

During the EM therapeutic session, following the initial live instrumental or vocal intervention (Step 8 of EM Active), the music therapist initiates iterative processes.

Horizontal Process

Each horizontal iteration represents a cycle wherein a single stimulus (simple or compound) is administered to the patient, a response is obtained, and the stimulus is updated for subsequent administration (Supplementary Figure 22).

Vertical Process

Vertical iterations involve the superimposition of multiple sound stimuli simultaneously, forming a complex set of stimuli to which the patient responds. This response is then used to create a new composite stimulus.

Structure and Key Concepts

I. Sound Archive (Categories from A_0 to A_9)

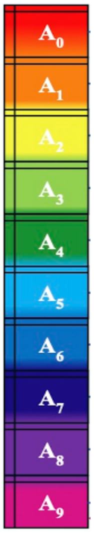

A_0: Therapist's Sound/Music

A_1: Family Sound

A_2: Music Known by the Patient

A_3: Discomfort Sound

A_4: Amniotic Sounds

A_5: Sound Design

A_6: Electronic Noise

A_7: Body Sound

A_8: Live Music and Instruments Sound

A_9: Patient Reaction

II. Sound Stimuli (S_n):
- S_1: Sound stimulus extracted from the archive.

III. Patient Reactions (R_n):
- R_1: First patient reaction.

IV. Compound Stimuli (Sc_n):
- Sc_1: Compound stimulus resulting from $S_1 + R_1$
- Sc_2: Compound stimulus resulting from $Sc_1 + R_2$
- and so on...

Horizontal Iterative Scheme

Iteration 1:

1. Administration of Sound Stimulus (S_1):

- S_1 is extracted from one of the categories A_0-A_9.

2. Patient Response (R_1):
   - The patient responds with R_1.

3. Formation of Compound Stimulus (Sc_1):
   - $Sc_1 = S_1 + R_1$

Iteration 2:

1. Administration of Compound Stimulus (Sc_1):
   - Sc_1 is administered to the patient.

2. Patient Response (R_2):
   - The patient responds with R_2.

3. Formation of Compound Stimulus (Sc_2):
   - $Sc_2 = Sc_1 + R_2$

Iteration 3:

1. Administration of Compound Stimulus (Sc_2):
   - Sc_2 is administered to the patient.

2. Patient Response (R_3):
   - The patient responds with R_3.

3. Formation of Compound Stimulus (Sc_3):
   - $Sc_3 = Sc_2 + R_3$

This schema can continue iteratively, updating the compound stimulus each time with the new patient response.

Vertical Iterative Schema: Overlaying Musical Tracks

1. Vertical Sound Stimuli (V_n):
   - Sound stimuli from different categories can be vertically overlaid.

2. Overlay of Stimuli:
   - $V_1 = \{S_1(A_0), S_2(A_1), S_3(A_2), ...\}$

3. Patient Response (RV_n):
   - The patient's response to V_1 vertical stimulus is RV_1.

4. Formation of Vertical Composite Stimulus (ScV_n):
   - $ScV_1 = V_1 + RV_1$

Example of Vertical Process

Vertical Iteration 1:

1. Administration of Vertical Stimuli (V_1):
   - $V_1 = \{S_1(A_0) + S_2(A_1) + S_3(A_2)\}$

2. Patient Response (RV_1):
   - The patient responds with RV_1.

3. Formation of Vertical Composite Stimulus (ScV_1):
   - $ScV_1 = V_1 + RV_1$

Vertical Iteration 2:

1. Administration of Vertical Stimuli (V_2):
   - $V_2 = \{ScV_1 + S_4(A_3)\}$

2. Patient Response (RV_2):
   - The patient responds with RV_2.

3. Formation of Vertical Composite Stimulus (ScV_2):
   - $ScV_2 = V_2 + RV_2$

Overall Schema: Horizontal and Vertical

Horizontal Iteration 1:

$S_1(A_0) \rightarrow R_1$

$Sc_1 = S_1 + R_1$

Horizontal Iteration 2:

$Sc_1 \rightarrow R_2$

$Sc_2 = Sc_1 + R_2$

Vertical Iteration 1:

$V_1 = \{S_1(A_0) + S_2(A_1) + S_3(A_2)\}$

RV_1

$ScV_1 = V_1 + RV_1$

Vertical Iteration 2:

$V_2 = \{ScV_1 + S_4(A_3)\}$

RV_2

$ScV_2 = V_2 + RV_2$

This integrated schema describes the rehabilitative process in EM music therapy using both the horizontal iterative approach, showing the temporal progression of patient responses, and the vertical

iterative approach, describing the simultaneous overlay of different sound stimuli. These approaches can be combined to create a dynamic and adaptable therapeutic program, optimized based on individual patient responses.

Supplementary Figure 22. EM Horizontal Iterative Schema of Stimuli and Patient's Responses.

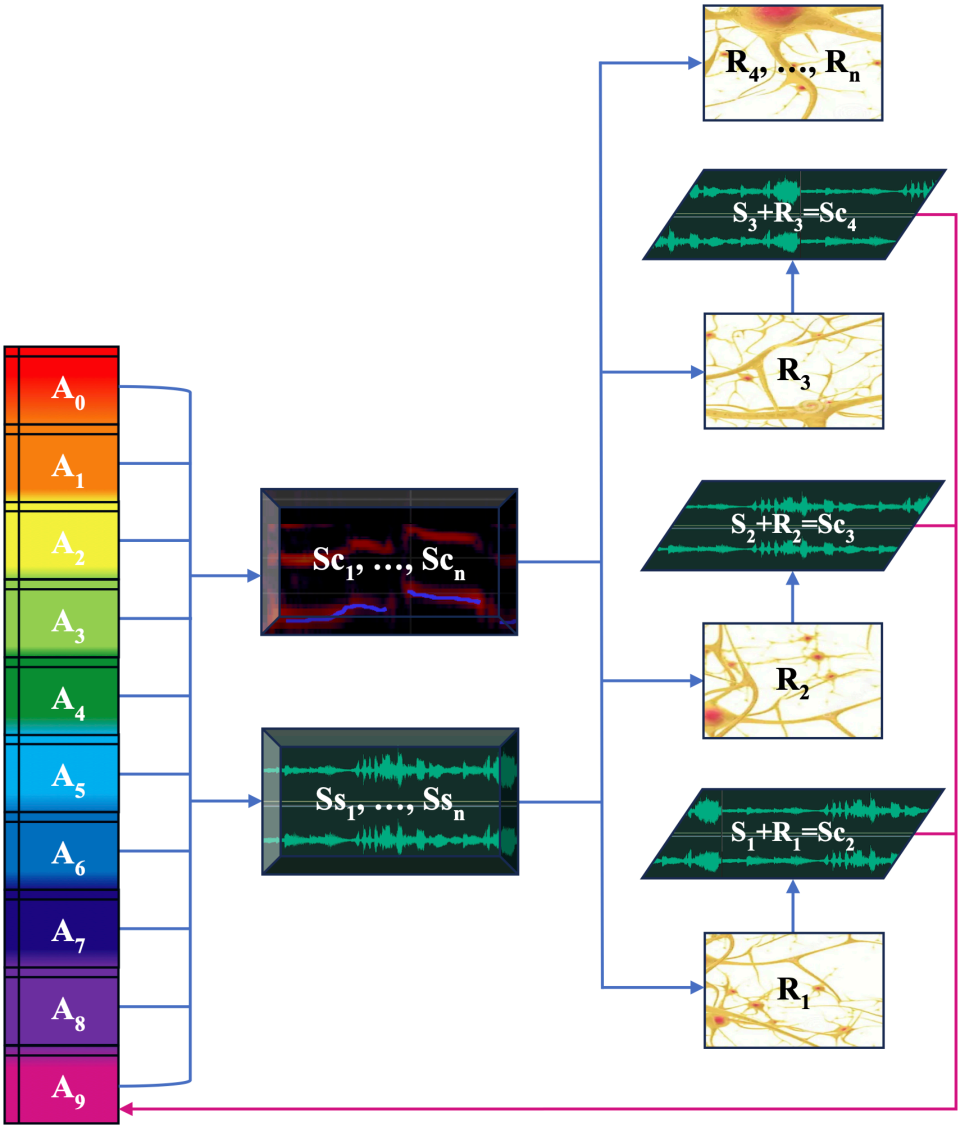

LEGEND

$A_{0..9}$ = Sound archive A

S_n = *Ss* or *Sc*

Ss_n = Simple sound stimuli

R_n = Patient's reactions

Sc_n = Compound sound stimulus

S4. Technical-Musical Analysis for the Composition of a Personalized Therapeutic Composition (PTC) and an Audiovisual Soundtrack (AVS).

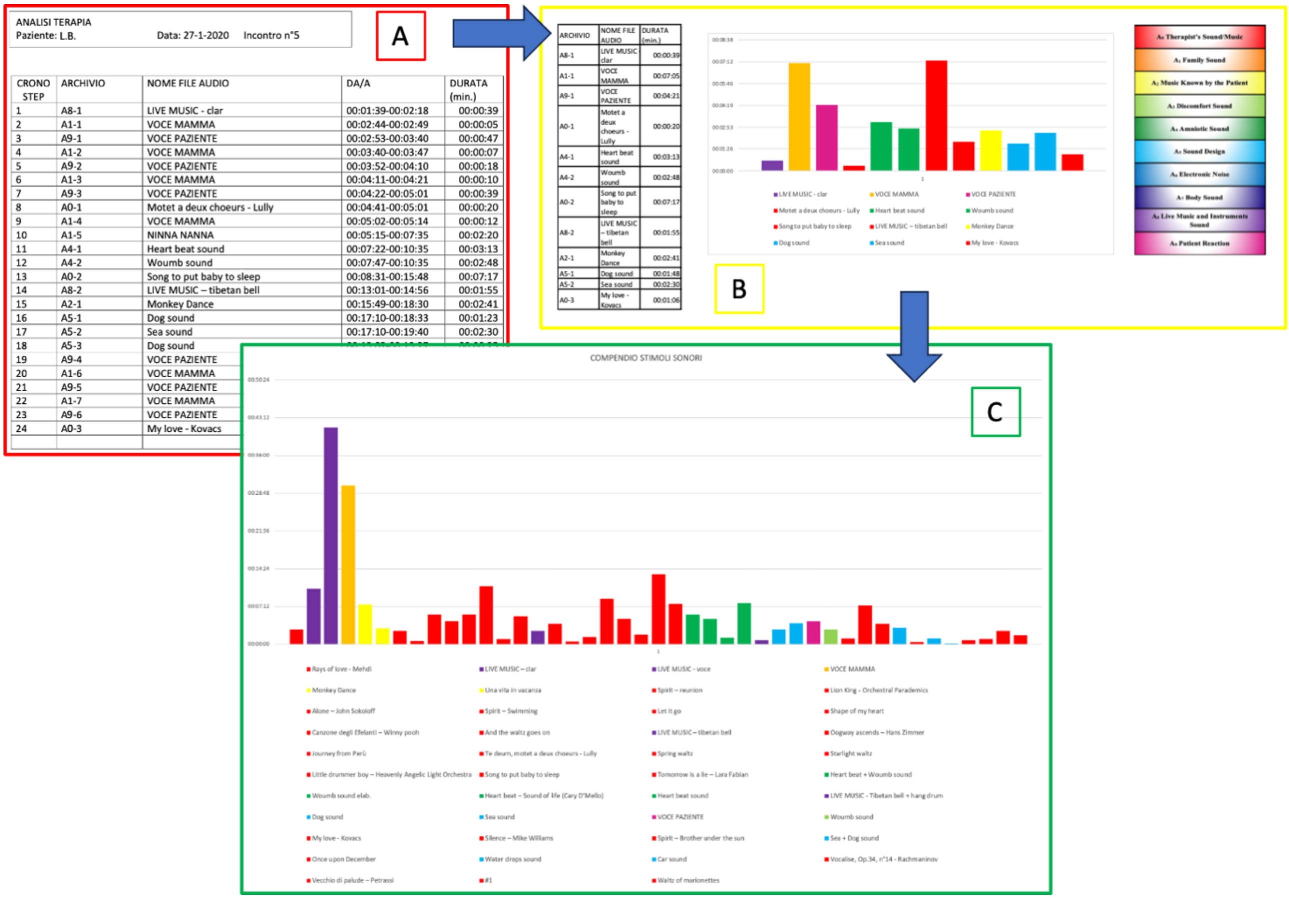

Supplementary Figure 34. Technical-musical analyses analysis.

Step A: Data Collection and Recording

1. Chronostep:

- Collect temporal data related to each significant sound event during therapy.
- Record the exact moment during the therapy session when the patient responds to the stimulus. This information is essential for analysis of reaction time and stimulus-response correlation.

2. Name audio files that are significant to the patient (stimulus type):

- Give each audio file a descriptive and unique name that represents the type of sound stimulus.
- Classify these sound stimuli according to their type and relevance to the patient.

3. Reference Archive:

- Specify the archive from which the stimulus was extracted (e.g., A0: therapist's sound/music, A1: music known to the patient, etc.).

4. Duration of each clip administered:

- Measure and record the duration of each audio clip used during therapy.
- Analyze how the duration of the stimulus affects the patient's response.

Step B: Analysis of Patient Responses

1. Export Patient Reaction Times to Excel:

- Export the patient response time data to an Excel spreadsheet for detailed analysis.
- Organize the data in a table with columns for audio file name, reference archive, time of administration, clip duration, and reaction time.

2. Transform the data into a histogram.

- Use the exported data to create a histogram that graphically represents the temporal distribution of the patient's responses. This allows you to easily visualize patterns and the time periods during which the patient's responses are most frequent or intense.

Step C: Identification of significant musical sections

1. Review the clips for the patient:

- Analyze the histogram to identify the musical sections that elicited the most significant responses.
- Select the audio clips corresponding to these moments to create a Personalized Therapeutic Composition (PTC).

Creating the PTC and Audiovisual Soundtrack (AVS)

1. Select significant audio clips for the PTC:

- Overlay the effective clips in the multitrack according to the "Hierarchical Perceptual Function" criterion to compose the PTC.

2. Integration of images and videos from the therapeutic journey:

- Collect the most relevant images and videos that document the patient's therapeutic journey.
- Integrate these visual elements with the PTC based on the sound-image correspondence criterion to create an audiovisual soundtrack (AVS) that represents the patient's progress and experience.
